# Tailoring Cathode–Electrolyte Interface for High-Power and Stable Lithium–Sulfur Batteries

**DOI:** 10.1007/s40820-024-01573-4

**Published:** 2024-12-04

**Authors:** Mengting Liu, Ling-Jiao Hu, Zhao-Kun Guan, Tian-Ling Chen, Xin-Yu Zhang, Shuai Sun, Ruoli Shi, Panpan Jing, Peng-Fei Wang

**Affiliations:** 1 Center of Nanomaterials for Renewable Energy, State Key Laboratory of Electrical Insulation and Power Equipment, School of Electrical Engineering, Xi’an Jiaotong University, Xi’an, 710049 People’s Republic of China; 2 Low-Dimensional Materials and Photo/Electrochemical Technology Lab, School of Materials Science and Engineering, Shaanxi Key Laboratory of Green Preparation and Functionalization for Inorganic Materials, Shaanxi University of Science & Technology, Xi’an, 710021 People’s Republic of China

**Keywords:** Lithium–sulfur batteries, Shuttle effect, Cathode–electrolyte interface, Structural enhancement, Reaction pathway

## Abstract

Global interest in lithium–sulfur batteries as one of the most promising energy storage technologies has been sparked by their low sulfur cathode cost, high gravimetric, volumetric energy densities, abundant resources, and environmental friendliness. However, their practical application is significantly impeded by several serious issues that arise at the cathode–electrolyte interface, such as interface structure degradation including the uneven deposition of Li_2_S, unstable cathode–electrolyte interphase (CEI) layer and intermediate polysulfide shuttle effect. Thus, an optimized cathode–electrolyte interface along with optimized electrodes is required for overall improvement. Herein, we comprehensively outline the challenges and corresponding strategies, including electrolyte optimization to create a dense CEI layer, regulating the Li_2_S deposition pattern, and inhibiting the shuttle effect with regard to the solid–liquid–solid pathway, the transformation from solid–liquid–solid to solid–solid pathway, and solid–solid pathway at the cathode–electrolyte interface. In order to spur more perceptive research and hasten the widespread use of lithium–sulfur batteries, viewpoints on designing a stable interface with a deep comprehension are also put forth.

## Highlights

- This review delves into the mechanism of the state-of-the-art lithium–sulfur batteries from a novel perspective of cathode–electrolyte interface.
- It provides extensive strategies to construct a stable cathode–electrolyte interphase layer and improve the uneven deposition of Li_2_S, enhancing the stability of the interface structure.
- It proposes an in-depth and comprehensive research on how to inhibit the shuttle effect at the cathode–electrolyte interface with regard to distinct reaction pathways.

## Graphical abstract

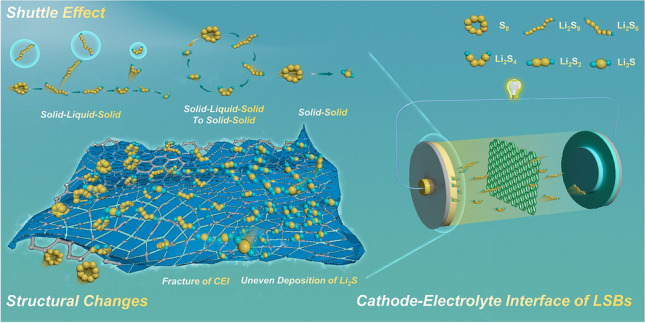

## Introduction

Continuously increased demand but lack of energy has emerged as one of the most pressing issues confronting human society since the second industrial revolution [1]. Energy storage technology has flourished as a result of the tremendous growth in green energy production to offset the overconsumption of traditional fossil fuels [2–8]. Electrochemical energy storage has brought about great breakthroughs from the grid to every aspect of human life. Due to the superiorities of significant energy density and long-term cycling stability, lithium-ion batteries (LIBs) have played a vital role in most electronic portable devices since their first commercialization in 1991 by Sony Corporation [9–15]. Nevertheless, the energy density of LIBs while once regarded as high compared to capacitors and lead-acid batteries can hardly keep up with the contemporary ever-increasing energy storage demands because the theoretical specific capacities of cathodes like LiFeO_4_, LiCoO_2,_ and LiMn_2_O_4_ are comparatively limited [16, 17]. Therefore, a number of energy storage alternatives “beyond LIBs” are investigated [18–21].

**Fig. 1:**
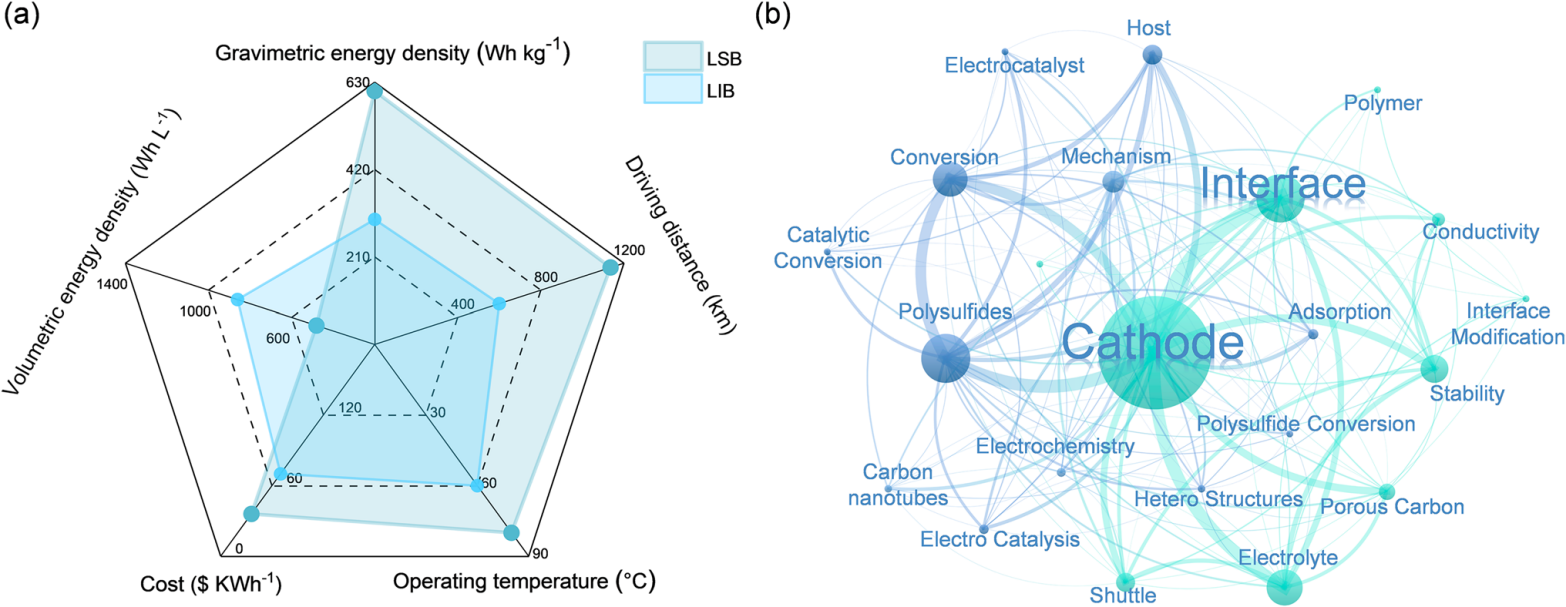
**a** Radar chart comparing key parameters of LSB and LIB [6, 22–25]. **b** Network of potential challenges and strategies of cathode–electrolyte interface throughout the reports of LIBs in the past decade

**Fig. 2:**
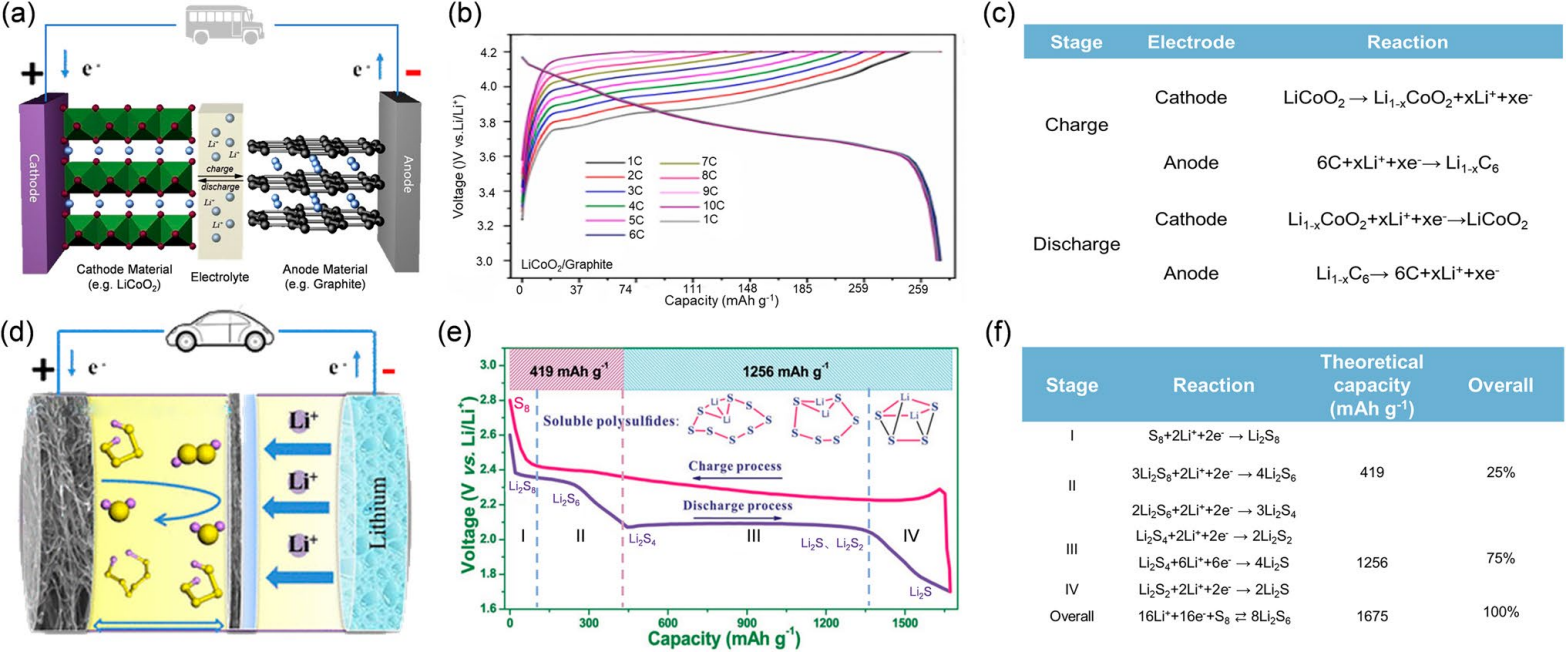
**a** Schematic internal configuration illustration of LIBs. Reproduced with permission from Ref. [71], Copyright 2011, Royal Society of Chemistry. **b** GCD curves of LiCoO_2_ cathode of LIBs. Reproduced with permission from Ref. [72], Copyright 2022, Royal Society of Chemistry. **c** Redox reactions of LiCoO_2_ cathode of LIBs [73]. **d** Schematic internal configuration illustration of LSBs. Reproduced with permission from Ref. [74], Copyright 2018, Royal Society of Chemistry. **e** Representative GCD curves of LSBs in the ether-based electrolyte. Reproduced with permission from Ref. [75], Copyright 2020, American Chemical Society. **f** Corresponding redox reactions of S cathode of LSBs. Reproduced with permission from Ref. [76], Copyright 2022, John Wiley and Sons

Lithium–sulfur batteries (LSBs) attracted widespread attention because of their potentially high theoretical energy density (2600 Wh kg^−1^) outperforming times the counterpart of conventional LIBs (LiCoO_4_:300 Wh kg^−1^) by approximately 8.6 [6, 26, 27]. As shown in Fig. 1a, the LSBs have wider operating temperature and much lower costs than LIBs. Moreover, together with the longer driving distance, the LSBs hold greater potential in commercial applications. Since LSBs and LIBs are both lithium-based batteries, the commercial application status of LSBs still could not compare with LIBs even regarding the unique merits of LSBs [28]. What blocks the application of LSBs requires deeper thinking and the underlying reason might trace back to its distinct working principle different from that of LIBs. The rocking chair-type battery like sodium-ion battery or LIBs mainly depends on the reverse intercalation and de-intercalation of Li^+^ from the cathode to anode during cycling and is therefore called a “rocking chair”-type battery [29–33]. Taking the LIB as an example with LiCoO_2_ cathode and graphite anode (Fig. 2a), the galvanostatic charge–discharge (GCD) curves and related chemical reactions that occur at electrodes can be presented in Fig. 2b, c, respectively. In comparison, the working principle of LSBs is much more complex and trickier, which not only has great differences in reactions at different stages but also involves complex solid–liquid–solid-phase revolution in conventional reaction pathway with two plateaus (Fig. 2e, f) [34–36]. The following are the specific reaction steps. The overall chemical reaction during the discharge process can be simplified as 16Li^+^ + S_8_ + 16e^−^ → 8Li_2_S, and it is a multi-step S reduction reaction. In stage I, S_8_ is first reduced to soluble Li_2_S_8_. In stage II at about 2.3 V (vs. Li^+^/Li), there comes the first plateau, attributed to the reduction process from Li_2_S_8_ to Li_2_S_6_. Afterward, the Li_2_S_6_ is reduced to Li_2_S_4_. The first two stages consist of solid–liquid-phase transformation contributing to a theoretic capacity of 419 mAh g^−1^ with 4 electrons [35]. Moreover, during the first two stages the formed reduction product Li_2_S_*x*_ (4 ≤ *x* ≤ 8) is all soluble and will dissolve into the electrolyte and shuttle back and forth between the cathode–electrolyte interface, separator, and the anode in the cycling process [25]. The dissolution in the first two stages is the origin of the notorious shuttle effect. In the subsequent stage III, the second plateau at about 2.1 V (vs. Li^+^/Li) corresponds to the liquid–solid-phase transformation from the Li_2_S_4_ to the Li_2_S_2_ and Li_2_S [37, 38]. In the last stage IV, the solid Li_2_S_2_ is eventually reduced to the solid Li_2_S. Compared with the first two stages, stage III and stage IV with 12 electrons transfer in total contribute a capacity of 1256 mAh g^−1^. Furthermore, it has been demonstrated that the deposition of Li_2_S and the speciation of S_8_ are both the rate-determining steps in the discharge and charge process, respectively, through the CV profiles [39, 40]. This sluggish kinetics might originate from its uneven deposition pattern and insolubility. Due to the density differences of Li_2_S and S, the volume of the cathode will also change drastically during cycling [41–43]. In other components of LSBs, the lithium dendrites growth, anode volume expansion, and unstable solid electrolyte interphase (SEI) layer pose a threat to the anode and the uncontrollable lithium dendrites might pierce through the separator [44–47]. It has been reported that the shuttling high-order intermediate polysulfides (LiPSs) could have parasitic reactions with the lithium anode and form the insualting layer of Li_2_S_2_ and Li_2_S to passivate and corrode the anode [48, 49]. All the issues mentioned above will cause the loss of active materials, low coulombic efficiency, capacity decline, and even safety hazards.

Over the past few years, extensive studies have been dedicated to mitigating the issues mentioned above. Firstly, various methods have been explored to enhance the conductivity of S cathodes [50]. The incorporating of conductive additives such as carbon nanotubes or graphene has proven to be an effective approach for improving electron transport [51]. In terms of the S host, researchers have favored the construction of porous framework with connected pores, effectively increasing electronic conductivity while providing a buffer space for volume expansion of S [52, 53]. Secondly, significant advancements have been made in separator technology to prevent the diffusion of LiPSs while enabling efficient ion diffusion [54, 55]. To date, three kinds of mainstream separators, including sandwiched, janus, and composite structure, have been employed to capture the LiPSs and enhance ionic conductivity [56–59]. Thirdly, as an indispensable part in the smooth function of the LSBs, lithium alloys and carbon-based materials have been widely investigated to address dendrite formation and low coulombic efficiency of anodes [45, 60, 61]. Moreover, constructing Li composites, artificial SEI layer, and additives in the electrolyte is also adopted to stabilize the Li metal anode [62–64]. Finally, since the composition and formulation of the electrolyte are crucial for achieving stable and high-performance LSBs, a variety of liquid and solid electrolytes have been explored, with a focus on optimizing parameters such as ionic conductivity, electrochemical stability, and compatibility with cathode and anode [65–67].

Therefore, LSBs have made long-term strides in the performance from the rational design and modifications of cathode, anode, and separator to the electrolyte optimization tactics. However, the electrode–electrolyte interface is hard to be neglected as the energy exchange position of the LSBs. The interfacial physicochemical properties and stability are closely linked to the comprehensive performance [65, 68]. Recent research has focused on understanding the interface behavior to acquire a profound insight into the electrochemistry in LSBs. It is clearly shown in Fig. 1b that the issues in the cathode–electrolyte interface are strongly associated with its structure as well as the dissolution and diffusion of LiPSs. Thus, it is essential to understand the mechanisms underlying the challenges at the cathode–electrolyte interface, as feasible and affordable strategies are urgently needed to fuel the further development of LSBs [69, 70].

In this review, we will take a deep look at the bottleneck challenges and the corresponding optimization strategies at the cathode–electrolyte interface of LSBs. The critical challenges are discussed from structural and shuttle effect, respectively. Various methods are proposed to regulate the formation of cathode–electrolyte interphase (CEI) layer and the deposition pattern of Li_2_S in order to enhance the structural stability. Moreover, comprehensive research is conducted to relieve the shuttle effect by restraining the LiPSs at the interface from three different reaction pathways. The limitations and possible future direction in manipulating the conductive and thermodynamically stable cathode–electrolyte interface to improve the durability of LSBs are also claimed.

## Challenges of Cathode–Electrolyte Interface

It is crucial to explore the origin of the challenges at the cathode–electrolyte interface before employing the strategies. It stems not only from the formation of the CEI layer but also from the conversion mechanism mentioned above. The CEI layer is generally formed at the interface during the first cycling. The deposition of Li_2_S and shuttle effect also takes place at the cathode–electrolyte interface. During cycling, the fracture of the CEI layer and uneven deposition of the Li_2_S take a heavy toll on the interface stability. The shuttle effect triggered by the dissolution of LiPSs at the interface requires urgent care for the high-power LSBs.

### Interface Structural Changes

#### Fractured CEI Layer

The CEI layer serves as a protective barrier at the cathode–electrolyte interface to safeguard the entire cathode and prevent direct contact between LiPSs and electrolyte [77, 78]. Understanding its formation mechanism and factors leading to instability of the CEI layer is crucial for enhancing the cathode–electrolyte interface. It is widely accepted that the formation of the CEI layer is closely associated with the deposition of electrolyte and oxidation of solvent molecules on the active cathode surface [79–83].

**Fig. 3:**
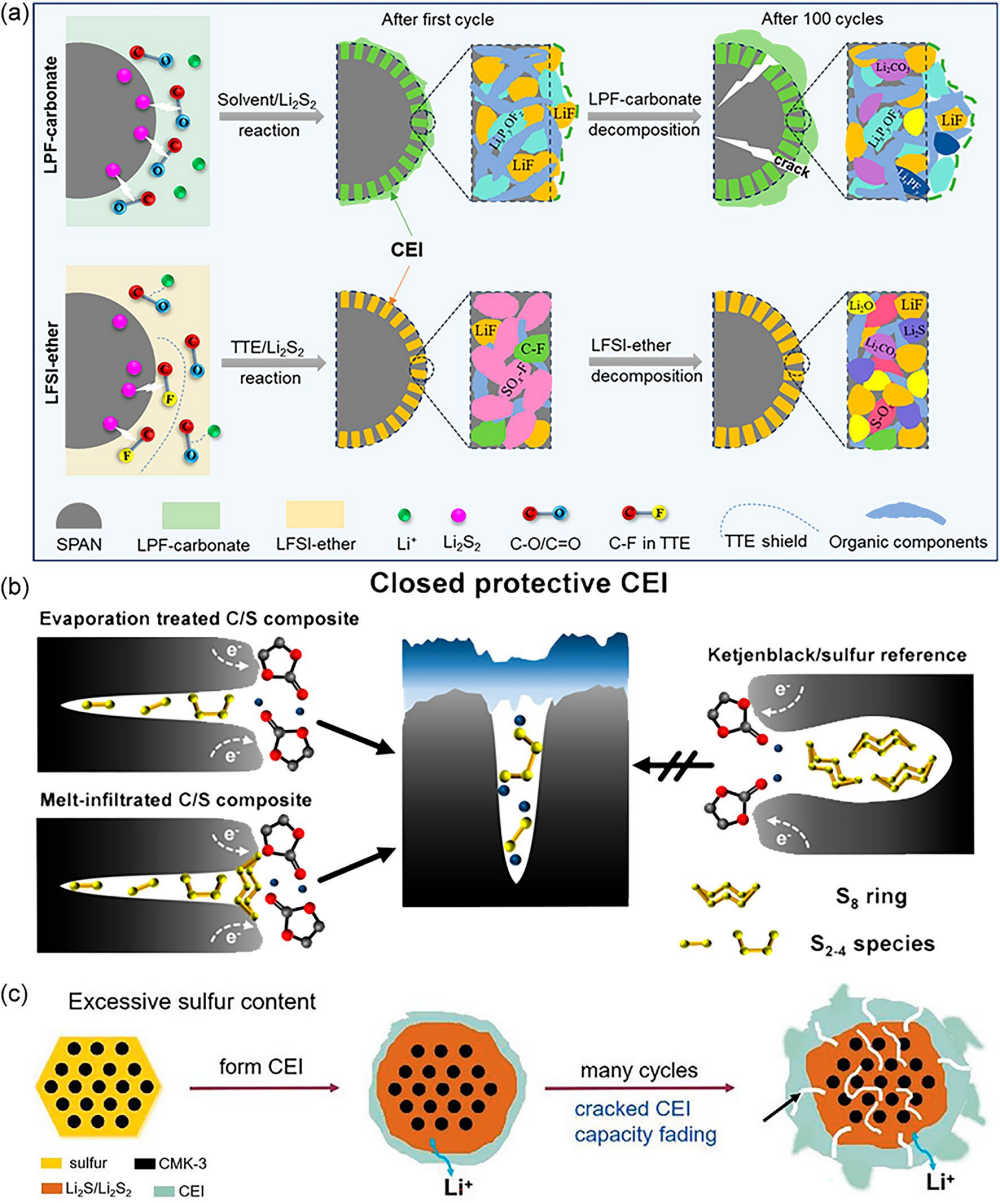
**a** Diagram of CEI formation mechanism and process for SPAN in LPF-carbonate and LFSI-ether electrolytes. Reproduced with permission from Ref. [86], Copyright 2022, American Chemical Society. **b** Schematic illustration of the evaporation treated, melt-infiltrated composite, and Ketjenblack/S cathode during initial discharge process of CEI formation. Reproduced with permission from Ref. [89], Copyright 2020, John Wiley and Sons. **c** Schematic illumination of fractured CEI layer with an excessive S content. Reproduced with permission from Ref. [90], Copyright 2022, John Wiley and Sons

In the case of LSBs, the formation mechanism of the CEI layer varies across different electrolytes (Fig. 3a) [84–86]. For instance, in a carbonate-based electrolyte system consisting of 1.0 M LiPF_6_ in ethylene carbonate (EC)/ethyl methyl carbonate (EMC) (3:7 by wt%) with 2 wt% vinylene carbonate (VC), the CEI layer is generated through the nucleophilic reaction between the C–O/C=O bonds and Li_2_S_2_, resulting in the formation of LPF-carbonate with C–S bonds and inducing further solvent decomposition. The primary components of the CEI layer are organic compounds. On the other hand, in an ether-based electrolyte containing lithium bis(fluorosulfonyl)imide (LiFSI), 1,2-dimethoxyethane (DME), 1,1,2,2-tetrafluoroethyl-2,2,3,3-tetrafluoropropyl ether (TTE) at the molar ratio of 1:1.2:3, DME solvent is hard to degrade because of the connect between its abundant C–O bonds and Li^+^. In contrast, the LiF is formed due to the high activity of Li_2_S_2_ to C–F bonds in TTE giving rise to the decomposition of the TTE. Moreover, the reaction between the LiFSI and Li_2_S_2_ will break the S–N bonds and generate SO_x_-F species. Thus, the CEI layer is dominated by the SO_x_-F species and the limited LiF in the ether-based electrolyte.

Actually, systematically understanding the formation, structure, composition as well as the tailored interphase chemistry of CEI layers, remains an ongoing research. The formation of the stable CEI layer in common electrolytes also poses challenges. In common ether electrolytes, the high solubility of LiPSs inhibits the formation of a solid-state CEI layer. Additionally, the continuity and densification of the CEI layer are negatively impacted by low-activity lithium salts like trifluoromethanesulfonimide (LiTFSI). Although the carbonates can facilitate the formation of a relatively dense CEI layer in common carbonate systems (e.g., EC:EMC = 1:2 with 1 M LiTFSI), the uninterrupted direct contact between carbonates and LiPSs allows for continuous reactions between LiPSs and C=O groups of the ester electrolyte, resulting in a poorly controlled thickness of the CEI layer, which may eventually lead to passivation of the cathode–electrolyte interface [87, 88]. Furthermore, the porosity of S host and S content also influences the formation and stability of the CEI layer. Excessive porosity of the host material and excessively small particles of the S may hinder the formation of a closed CEI layer (Fig. 3b) [89]. When the S content is too high, the formed CEI layer may fracture due to the inability to withstand mechanical stress, owing to the difference in density between S and Li_2_S (Fig. 3c) [90].

#### Uneven Li_2_S Deposition

**Fig. 4:**
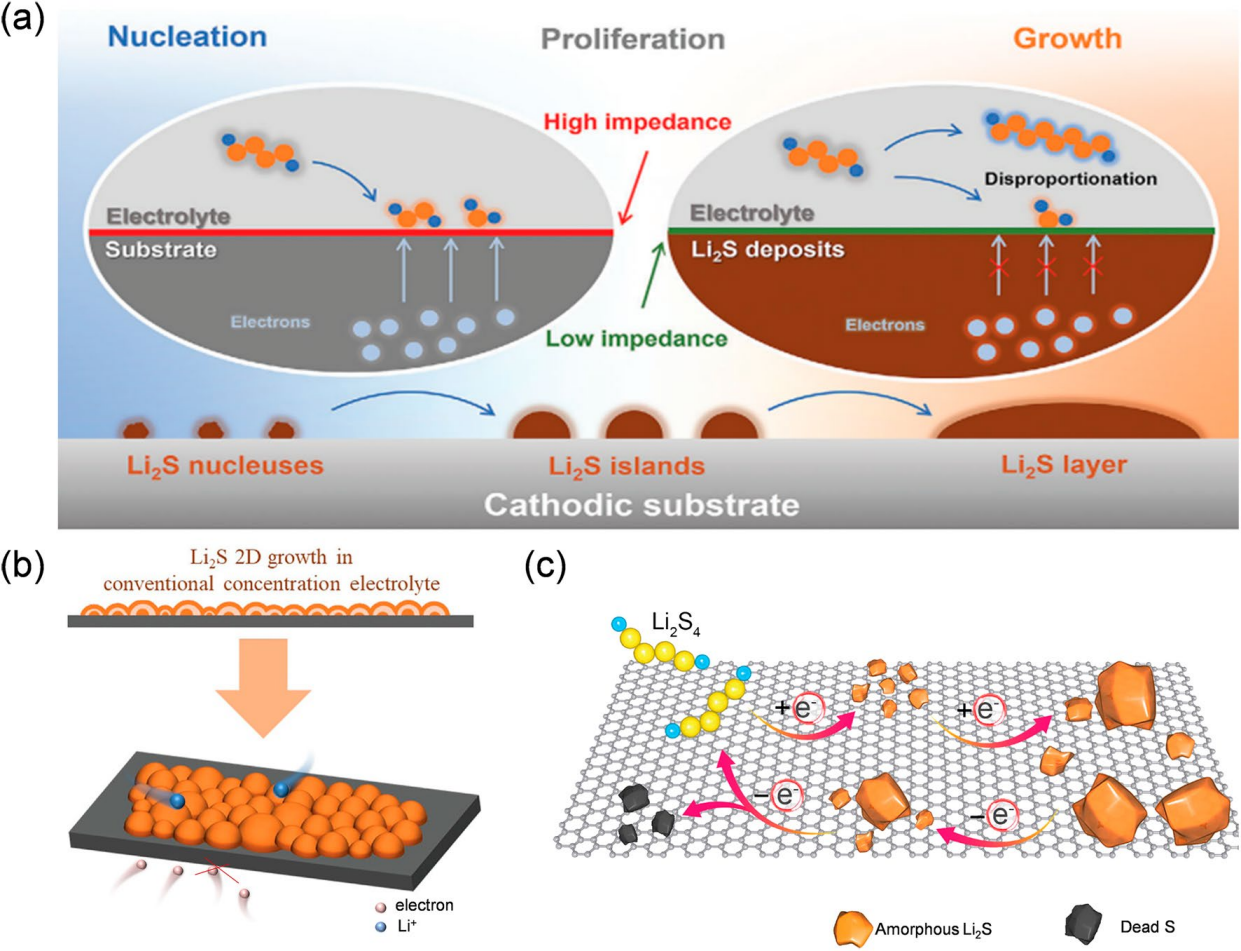
**a** The “nucleation–proliferation–growth” model of Li_2_S. Reproduced with permission from Ref. [91], Copyright 2023, John Wiley and Sons. **b** Illustration of Li_2_S 2D growth. Reproduced with permission from Ref. [95], Copyright 2023, John Wiley and Sons. **c** Deposition of Li_2_S on non-electrocatalytic surface. Reproduced with permission from Ref. [98], Copyright 2022, Elsevier

The deposition of the insulating product Li_2_S at the cathode–electrolyte interface is generally thought to be the rate-determining stage during the discharge process of LSBs. It is necessary to explore its mechanism before taking further action to improve the reaction kinetics of the LSBs. The deposition of Li_2_S is commonly presented as a nucleation–proliferation–growth model (Fig. 4a) [91].

Firstly, the Li_2_S nucleates on the cathode substrate by overcoming a high interfacial impedance [92]. As-formed nucleates typically precipitate as islands after merging with LiPSs at the cathode–electrolyte interface driven by an electrochemical process, where the impedance between similar species is rather low [93]. Subsequently, the insulating Li_2_S islands will keep expanding and eventually proliferate into a layer at the interface, which will hinder the electron transfer at the S host interface, passivating the cathode and decreasing S utilization [94]. As vividly shown in Fig. 4b**,** the conventional 2D growth will lead to the uneven deposition pattern of insulating Li_2_S [95]. However, the Li_2_S can continuously come into formation with limited electrons though unevenly deposited. The underlying reasons could be diverse. It has been proposed that Li_2_S_4_ can disproportionately form Li_2_S_7_ and Li_2_S in the absence of electrons [96, 97]. There exists a possible reaction pathway whereby the Li_2_S layer can persist in growing when the electrons can hardly transfer through the as-formed layer. This might further result in problems. Furthermore, it has been found that the morphology of the Li_2_S is connected to the electrocatalytic characteristics of the deposition surface. At the electrocatalytic surface, the crystalline and spherical Li_2_S is observed, while on a regular conductive surface, the amorphous and irregular Li_2_S is deposited unevenly [98]. It is probably due to the limited electron access and part of the thick Li_2_S layer could not be oxidized, thus leading to the uneven deposition of Li_2_S, or what is known as “dead Li_2_S” as shown in Fig. 4c. Essentially, such uneven Li_2_S deposition on the cathode will function as an insulating layer and passivate the cathode–electrolyte interface. It can even block pores of the host and impede electrons/ions diffusion, leading to sluggish reaction kinetics, an increase in interfacial impedance, and rapid capacity decay. Meanwhile, the irregular Li_2_S layer may also damage the structure of the cathode–electrolyte interface and influence the homogeneous reaction of the subsequent sites.

### Shuttle Effect

**Fig. 5:**
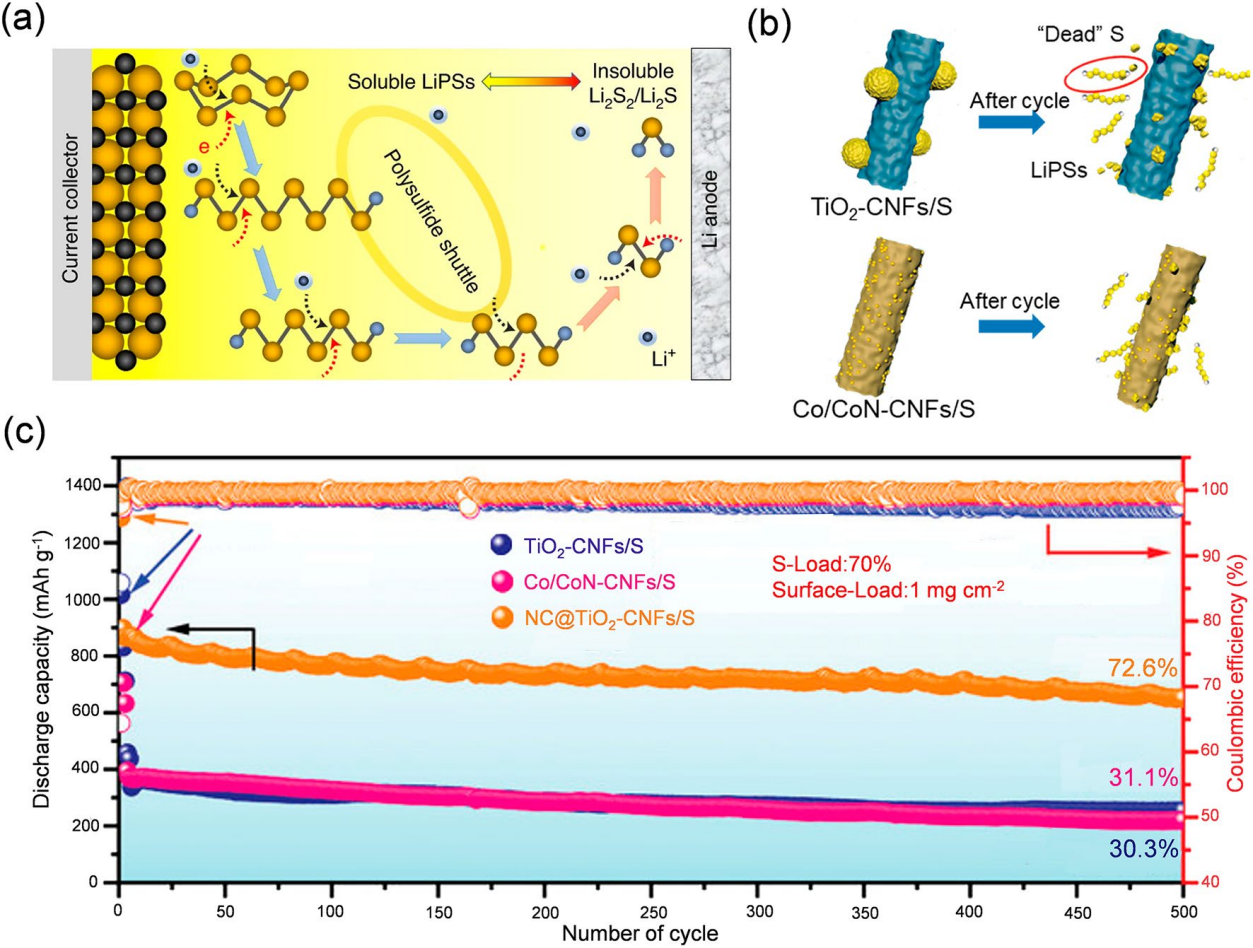
**a** Schematic illustration of shuttle effect. Reproduced with permission from Ref. [99], Copyright 2020, Springer Nature. **b** Schematic illustration of examples of improper hosts like TiO_2_-carbon nanofibers (CNFs)/S and Co/CoN-CNFs/S causing shuttle effect. **c** Cycling performance with TiO_2_-CNFs/S, Co/CoN-CNFs/S, and NC@TiO_2_-CNFs/S as host, respectively. **b, c** Reproduced with permission from Ref. [102], Copyright 2023, John Wiley and Sons

The multi-step conversion of long-chain LiPSs generated at the cathode–electrolyte interface in the LSBs always results in more serious problems because of their severe solubility in most common ether-based electrolytes. The process of the shuttle effect can be divided into the following stages (Fig. 5a). (1) The solid S_8_ is reduced to long-chain soluble LiPSs at the cathode–electrolyte interface. (2) The LiPSs detach from the S host and diffuse into the electrolyte. (3) The dissolved LiPSs shuttle to the anode side and have side reactions with the lithium, leading to the partial loss of the active materials and impeding the reaction kinetics. (4) In the charging process, the LiPSs will shuttle back to the cathode under the action of electric field force and have a disproportionation reaction with S_8,_ to form soluble Li_2_S_6_ and Li_2_S_8_, which further intensifies the loss of the active materials and deteriorates the structure of the S cathode [99–101]. For instance, compared to the proper host NC@TiO_2_-CNFs/S, other hosts like TiO_2_-CNFs/S and Co/CoN-CNFs/S cannot inhibit the shuttle effect and this results in the woeful degradation of active S accompanied with irreversible capacity loss and extreme decrease in coulombic efficiency as illustrated in Fig. 5b, c, plaguing the wide-scale application of LSBs [102].

Understanding the origins of challenges at the cathode–electrolyte interface is of great importance. On the one hand, the fractured CEI layer due to the volume change of cathode and uneven deposition of Li_2_S can damage the interface structure. On the other hand, the dissolution of long-chain LiPSs at the cathode–electrolyte interface will not only cause the constant loss of active materials from the S cathode but also cause the electrolyte to become more viscous. However, the systematic and more profound research on these thorny problems is worthy of more attention to provide a novel specific solution perspective.

## Interface Tailoring Strategies

In order to address the aforementioned thorny issues of cathode–electrolyte interface to achieve efficient Li^+^ diffusion kinetics for LSBs, significant strategies have been developed for tailoring the interface structure and suppressing the shuttle effect.

### Interface Structural Tailoring

#### Structural Enhancement of CEI Layer

The development of the CEI layer is closely linked to the cathode and the electrolyte. To ensure a uniform and dense CEI layer, it is crucial to manage cathode volume expansion and optimize the electrolyte composition [103].

**Fig. 6:**
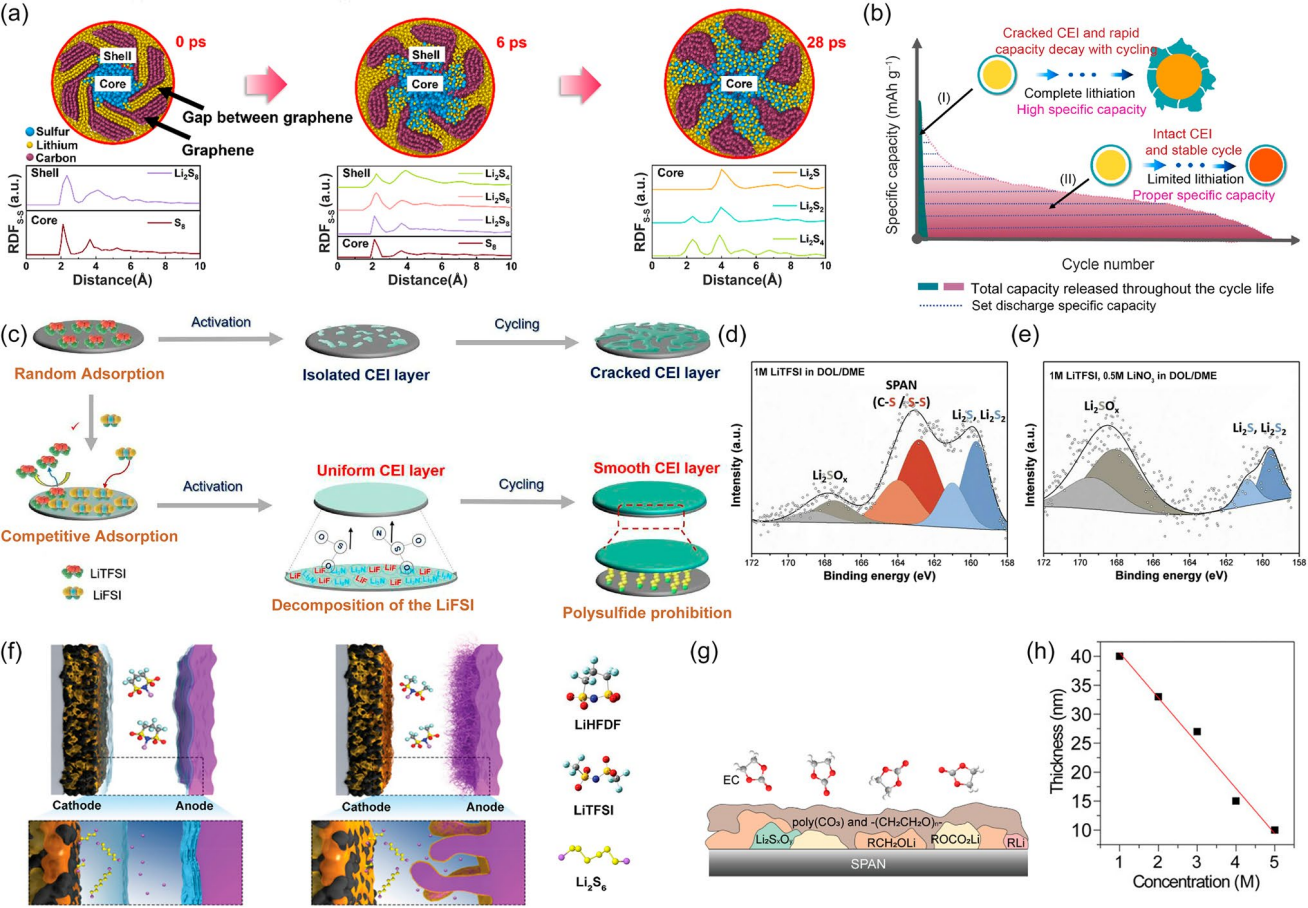
**a** Lithiation of the S with graphene hosts and RDFs of S–S atom pairs of lithiation of the S with graphene hosts at different times. Reproduced with permission from Ref. [43], Copyright 2024, Elsevier. **b** Schematic illustration of the fundamental functions of capacity control on the cycle life evolution of cells. Reproduced with permission from Ref. [106], Copyright 2022, Elsevier. **c** TFSI^−^ and FSI^−^ anion on FeS_2_@3DNPC electrodes. Reproduced with permission from Ref. [112], Copyright 2022, Elsevier. XPS S 2*p* spectra of polyacrylonitrile (SPAN) cathode cycled in **d** 1 M LiTFSI and **e** 1 M LiTFSI-0.5 M LiNO_3_ in 1,3-dioxolane (DOL)/1,2-dimethoxyethane (DME). **d**, **e** Reproduced with permission from Ref. [109], Copyright 2019, Elsevier. **f** The schematic illustration of how LiHFDF suppresses dissolution/shuttling of LSBs. Reproduced with permission from Ref. [113], Copyright 2020, John Wiley and Sons. **g** Schematic structure component of CEI layer formed in 1 M LiFSI/DME-EC. Reproduced with permission from Ref. [114], Copyright 2021, American Chemical Society. **h** The relationship between the thickness of the CEI layer and LiTFSI concentration. Reproduced with permission from Ref. [115], Copyright 2023, John Wiley and Sons

Previous studies have demonstrated that the CEI layer rupture caused by cathode volume expansion could be restrained by applying a suitable matrix host. Nanocarbon materials possessing abundant pores and good flexibility are typically exploited to load S. For instance, graphene matrix can effectively mitigate the S volume expansion because its many inner gaps could guide S lithiation along its open ends (Fig. 6a) [43]. Li et al. designed a molybdenum carbide decorated N-doped carbon hierarchical double-shelled hollow spheres (N–C HDS-HSs) electrode [104]. A buffer space for S expansion was provided by the double-shell hollow structure, while the thick mesoporous inner shell and central voids significantly increased the loading content of S. Thus, the electrode performed an ultra-high cycling stability with a capacity of 1075.1 mAh g^−1^ and a retention rate of 96.3% after 100 cycles at 0.2 C. Chen et al. found that the CEI layer was intolerant to the volume change and fractured during repeated lithiation/de-lithiation when the volume of the reduction product (Li_2_S/Li_2_S_2_) surpassed the maximum volume of the host [90]. When the discharge becomes deeper, moreover, the volume expansion of Li_2_S increases and probably causes the instability and cracking of the CEI layer. Therefore, adjusting the depth of discharge by rationally managing the battery capacity and other discharge conditions is another effective way to safeguard the CEI layer (Fig. 6b) [105, 106]. One work reported that when the discharge specific capacity was limited to 300 mAh g^−1^ per cycle, the battery with an S loading of 4.56 mg cm^−2^ maintained a stable CEI layer and had a cycle life of more than 950 cycles with a capacity of 289 Ah g^−1^ over the course of its entire life.

Lithium salts, as the cornerstones of electrolytes, play a pivotal role in the formation of the CEI layer. As the commonly used lithium salt, the breakdown of anionic TFSI^−^ allows a certain quantity of LiTFSI molecules to be uniformly and smoothly attached to the cathode surface, forming a CEI layer rich in LiF^−^. With a high mechanical strength and ability to withstand the electrode volume change, the inorganic-rich CEI layer improves the electrochemical stability and suppresses irreversible reactions of electrolyte [107, 108]. Yet, a dense and continuous CEI layer is hardly obtained by utilizing LiTFSI alone due to the low activity of TFSI^−^ (Fig. 6c). It is impressive how adding co-salts can alleviate this problem. Concentrated ether-based electrolytes containing LiTFSI and LiNO_3_ can promote the formation of a CEI layer consisting of LiF and LiNO_2_ [109]. Meanwhile, the detection of the Li_2_SO_x_ component in the CEI layer demonstrated that the Li_2_S/Li_2_S_2_ was oxidized by LiNO_3_ (Fig. 6d, e). Li_2_SO_x_ has a stronger conductivity than Li_2_S/Li_2_S_2_ and contributes to constructing a sturdy solid-state CEI layer, which minimizes the direct exposure of discharge products to the electrolyte and so limits the generation and dissolution of soluble LiPSs. Also, the decreasing Li_2_S/Li_2_S_2_ on the cathode surface sharply decreases the cathode passivation. Previous studies have shown that LiFSI salt is unfavorable to LSBs over extended cycles because it has a high activity to react with LiPSs irreversibly, leading to S depletion [110]. However, it has been found recently that rational utilization of its high activity is beneficial for the formation of dense CEI layers containing large amounts of LiF-Li_3_N (Fig. 6c) [111, 112]. Additionally, reducing solvent adsorption on the cathode surface, lowering the Li^+^ desolvation barrier, and generating more free Li^+^ ions for fast transition kinetics were all made possible by the CEI layer, which provided an exceptional performance for LSBs. Satisfactory effects were also demonstrated by other highly active lithium salts with comparable decomposition components. The use of lithium 1,1,2,2,3,3-hexafluoropropane-1,3-disulfonimide (LiHFDF) results in a persistent physical barrier made up of LiF and Li_3_N by its cyclic and highly fluorinated anions construct, which confines LiPSs in the cathode bulk (Fig. 6f) [113]. With the S loading is 8.36 mg cm^−2^, the battery performed an initial area capacity of 7.49 mAh cm^−2^ (≈ 896 mAh g^−1^) and the capacity drops to 3.86 mAh cm^−2^ after 110 cycles. In the LiTFSI electrolyte, in contrast, the area capacity rapidly decreased to 1.5 mAh cm^−2^ after only 40 cycles from initial 7.65 mAh cm^−2^ (≈ 811 mAh g^−1^) with a S loading of 9.43 mg cm^−2^.

Dynamic regulation of the CEI layer through electrolyte modification is another effective initiative to preserve a stable cathode–electrolyte interface. A conformal polycarbonate-CEI layer, for example, can be induced at the interface when ethylene carbonate (EC) is designed as a co-solvent in ether electrolyte [114]. This layer is dominated by the organic (i.e., poly(CO_3_) and −(CH_2_CH_2_O)_n_−) (Fig. 6g). The increase in organic composition in a dense CEI layer enables to eliminate the LiPSs leakage and alleviate the electrolyte decomposition over time, thus achieving the thickness self-control. Also, the continuous presence of EC components in the electrolyte enables the repair of the highly variable CEI layer and keeps it dense. Additionally, the electrolyte modification notably facilitates the favorable role of LiTFSI for the formation of the CEI layer. Chang et al. further demonstrated that the TFSI^−^ was critical for the formation of a thin and dense CEI layer when VC served as the solvent [115]. As depicted in Fig. 6h, the thickness of the CEI layer varied with varying TFSI^−^ concentrations, most likely as a result of direct changes in the solvation structure and solvent activity of Li^+^ caused by the concentration of LiTFSI in electrolytes. Stated differently, TFSI^−^ may quantitatively regulate the structure of the CEI layer by controlling the electrolyte concentration.

#### Deposition Improvement of Li_2_S

Li_2_S tends to grow uncontrollably on the cathode surface as an insulating product in LSBs during the discharge process, which affects the conversion of LiPSs by passivating the cathode surface and hindering the transfer of electrons/ions. Therefore, Li_2_S deposition has to be improved. Generally, there are two means to that end. One is to achieve high Li_2_S solubility, and the other is to induce Li_2_S uniform deposition.

**Fig. 7:**
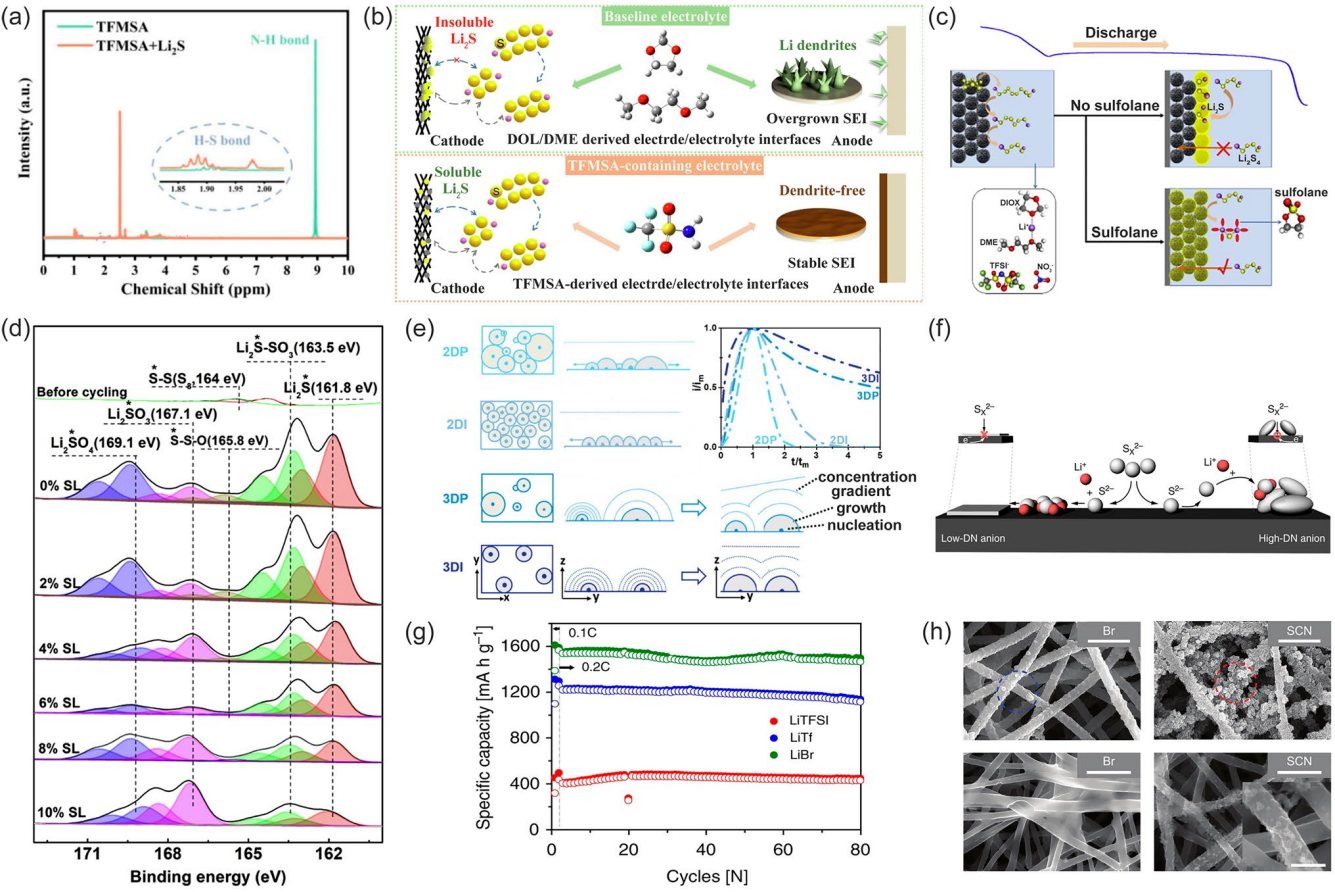
**a** Hydrogen NMR spectra of TFMSA and TFMSA + Li_2_S. **b** Positive effects of the TFMSA additive at electrode–electrolyte interfaces in the LSBs. **a**, **b** Reproduced with permission from Ref. [117], Copyright 2022, Elsevier. **c** Schematic diagram of enhancement effect of SL adding. **d** High-resolution S 2*p* XPS spectra of cathode surface with SL in 1 M LiTFSI in DOL/DME with 0.2 M LiNO_3_ containing different content of SL. **c**, **d** Reproduced with permission from Ref. [118], Copyright 2020, Elsevier. **e** Schematic illustration of 2D progressive nucleation (2DP)/2D instantaneous (2DI) (BFT models) and 3D progressive (3DP)/3D instantaneous (3DI) (SH models) (*x*–*y* is parallel to the substrate; *y*–*z* is vertical to the substrate). Reproduced with permission from Ref. [119], Copyright 2019, John Wiley and Sons. **f** Schematic diagram of 3D growth of Li_2_S induced by high donor number (DN) anions. **g** Comparison of the charge and discharge capacities for 80 charge/discharge cycles at 0.2 C. The electrolyte consists of 0.2 M LiPSs, based on Li_2_S_8_ and 1 M lithium salt Li_X_, *X* = TFSI^−^, Tf^−^, or Br^−^/0.2 M LiNO_3_/DOL: DME (1:1). **f**, **g** Reproduced with permission from Ref. [120], Copyright 2019, Springer Nature. **h** Li_2_S deposition morphologies in the cathode for the LiTFSI, LiBr, and LiSCN electrolytes after discharge at 0.05 C and 0.4 C. Reproduced with permission from Ref. [121], Copyright 2023, John Wiley and Sons

Strong interaction between the N–H bond and S^2−^ anion can access high Li_2_S solubility. The high electropositivity of the H atom in the N–H bond in ammonium salt, like NH_4_TFSI, can serve as a hydrogen bond donor to form H–S bonds with S^2−^, which in turn facilitates the dissociation of Li_2_S [116]. Since NH_4_TSFI promotes the high solubility of Li_2_S through the solvation process, its addition to the electrolyte can significantly reduce particle aggregation on the surface of the S/carbon tubes (CNT) cathode. In contrast with Li_2_S, the binding energy of soluble S^2−^ substance can be electrostatically stabilized by Li^+^, thereby facilitating the electrochemical S redox reaction. A similar behavior was also noted once the trifluoromethane sulfonamide (TFMSA) was added to the electrolyte, in which the S^2−^ in Li_2_S and the amide hydrogen (N–H) engaged in strong interactions to form the H–S bonds (Fig. 7a) [117]. The dissolution of Li_2_S at the interface produced more reaction sites for LiPSs conversion, accelerated the reaction kinetics, and raised the efficiency of the active S even with 1% TFMSA addition (Fig. 7b). Another approach for Li_2_S dissolution is the Lewis acid–base principle. Owing to the stronger Lewis basicity of Li_2_S compared to LiPSs, the solubilization of Li_2_S is facilitated by sulfolane (SL) as a Lewis acid, which is capable of strongly interacting with Li_2_S (Fig. 7c) [118]. As shown in Fig. 7d, the corresponding peak of Li_2_S drops dramatically (161.8 eV) with the increase in SL content in the electrolyte. At S loading of 1.0 mg cm^−2^, the cells containing 6% and 10% SL in 1 M LiTFSI in DOL/DME with 0.2 M LiNO_3_ displayed a promising capacity of 1130 mAh g^−1^ and 1050 mAh g^−1^, respectively, which were higher than that of the cells without SL additive in the electrolyte (1020 mAh g^−1^).

Li_2_S deposition on the cathode electrode surface follows an electrochemical deposition model (Fig. 7e) [119]. Generally, 2D Li_2_S deposition is a major obstacle to achieving high reversible capacity in the glyme-based LSBs as it leads to rapid loss of active electrode surface and low S utilization. Conversely, 3D deposition is capable of mediating the radial growth of Li_2_S, circumventing 2D laminar deposition and thus delaying electrode surface passivation. An effective strategy to achieve 3D growth of Li_2_S is electrolyte-based operation. It is well established that DN of solvent affects the deposition pattern of Li_2_S on the cathode surface. The passivation caused by uncontrollable Li_2_S and the 3D growth can be encouraged by high DN solvents. Chu et al. designed electrolytes with high DN anionic lithium salts, such as lithium triflate (LiTf) and LiBr [120] Tf^−^ and Br^−^ have both potent solvation effects on Li^+^ compounds, which can dissociate S^2−^ and increase the solubility of Li_2_S on the cathode surface. When S^2−^ that has left the electrode surface combines with Li^+^, Li_2_S will be deposited on the top surface nearby agglomerates due to its high polarity and then form a 3D structure (Fig. 7f). It significantly suppresses cathode interface passivation, prolongs the lower voltage plateau, and increases the discharge capacity to almost the theoretical value (Fig. 7g). Similar results are obtained from a novel thiocyanate anion (SCN^−^) salt with a high donor number (DN = 25.6 kcal mol^−1^). The dissociation of Li_2_S is facilitated not only by the strong coordination between SCN^−^ and Li^+^ but also by the direct interaction between SCN^−^ and S^2−^ [121]. Meanwhile, the short-chain LiPSs could be stabilized by electron-accepting C atoms in SCN^−^, providing more chemical pathways for Li_2_S deposition than those in the Br^−^ (Fig. 7h).

**Fig. 8:**
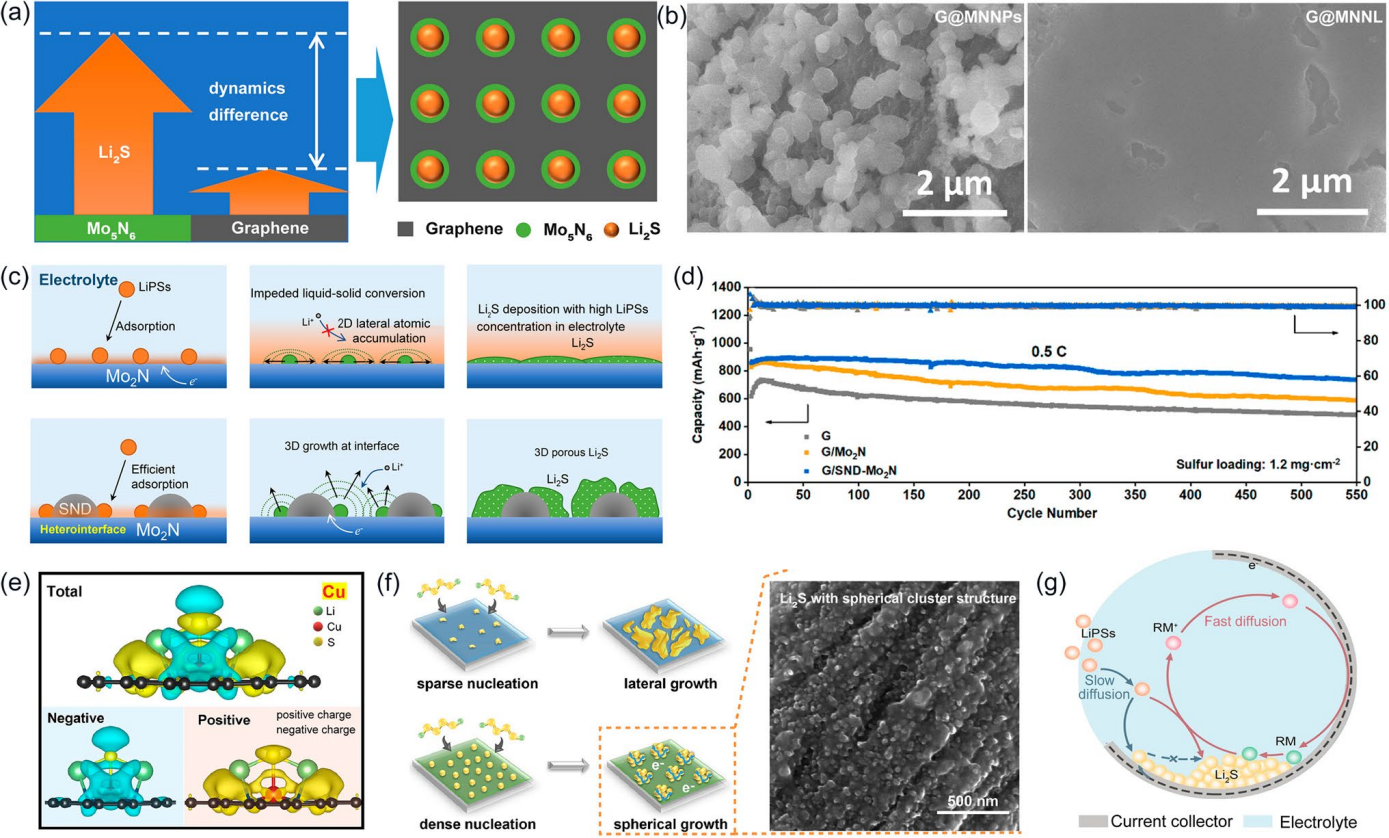
**a** Schematic illustration of the nucleation behavior of Li_2_S on G@MNNPs and **b** SEM images of G@MNNPs and G@MNNL after potentiostatic 5000 s discharge. **a**, **b** Reproduced with permission from Ref. [122], Copyright 2021, John Wiley and Sons. **c** Schematic conversions from LiPSs to Li_2_S on the Mo_2_N and SND-Mo_2_N surfaces, respectively, and **d** their long-term cyclability for 550 cycles at 0.5 C. **c**, **d** Reproduced with permission from Ref. [123], Copyright 2021, American Chemical Society. **e** Charge density difference of SA-Cu@N-doped graphene (NG)/Li_2_S. **f** Schematic illustrations of Li_2_S deposition process on CNF (top) and N-doped carbon fiber foam (SA-Cu@NCNF) (bottom) substrates. **e**, **f** Reproduced with permission from Ref. [124], Copyright 2022, Elsevier. **g** Schematic illustration of the growing pathway of Li_2_S in the absence (blue arrows) and presence (red arrows) of cobaltocene (CoCp_2_). Reproduced with permission from Ref. [125], Copyright 2019, John Wiley and Sons

In addition to electrolyte additives, metal-based materials have important applications in mediating the 3D deposition of Li_2_S because they provide a large number of reaction sites and improve the kinetic transformation process of S-containing materials. Tian et al. synthesized a composite host material of discrete Mo_5_N_6_ nanoparticles immobilized on graphene (G@MNNP) [122]. Because of its high catalytic activity, the Mo_5_N_6_ nanoparticles acted as favorable nucleation sites and guided isolated growth of the Li_2_S at the cathode-side interface (Fig. 8a). Isolated growth retarded the merging of neighboring Li_2_S nucleus and promoted their isotropic growth, and then, the 3D Li_2_S evolution was facilitated. As shown in Fig. 8b**,** solid deposits on G@MNNPs consisted of isolated Li_2_S following 5000 s of constant potential discharge. In contrast, the solid products on Mo_5_N_6_ nanolayer-coated graphene (G@MNNL) have relatively smooth morphology, indicating that Li_2_S almost entirely coverage the surface. A similar result was seen in the SnO_2_ nanodot (SND) modified Mo_2_N microstrip [123]. In comparison with the bare Mo_2_N, the SND/Mo_2_N heterointerface prevented the surface passivation of the Mo_2_N microstrip by facilitating the LiPSs adsorption and directing the 3D porous growth of Li_2_S (Fig. 8c). The battery reached a capacity as high as 738.3 mAh g^−1^ after 550 cycles at 0.5 C, and its decay rate was only 0.025% per cycle (Fig. 8d).

It should be noted that the insulating Li_2_S covering the catalytic site’s surface will diminish the catalytic activity, which in turn impacts the deposition efficiency. Single-atom copper modified SA-Cu@NCNF can effectively resolve this issue as a host material [124]. SA-Cu draws effective charge “acceptance–donation” between Cu and S due to strong metal-substrate interactions, enabling Li_2_S molecules to exhibit metal abundance with enhanced electronic conductivity (Fig. 8e). Assisted by the conducting Li_2_S clusters, the SA-Cu sites covered by Li_2_S clusters still serve as active sites for electrochemical reactions to further catalyze the 3D deposition of Li_2_S (Fig. 8f). Consequently, the SA-Cu@NCNF/S electrode exhibited a decay rate of 0.038% per cycle at 5 C after 500 cycles. One more effective tactic is to use soluble redox mediators. As an exogenous redox mediator formed by on-surface electroreduction, CoCp_2_ diffuses to the outer surface of pre-existing Li_2_S nuclei at the electrolyte/conducting substrate/Li_2_S triple-phase boundary and mediates Li_2_S growth (Fig. 8g) [125]. The CoCp_2_ always remained soluble during the catalytic process preventing changes in the amount and concentration caused by Li_2_S deposition, which continuously maintained the 3D Li_2_S growth. The discharge capacity of LSBs enhanced at least 8.1 times under harsh conditions like high multiplicity (> 1 C) or low electrolyte operation (electrolyte/S ratio of 4.7 uL mg^ −1^).

Additives featuring high dielectric constant, high viscosity, and appropriate DN are bound to improve the interface passivation due to the uneven deposition of Li_2_S and can construct a dense, uniform, and stable interface with high activity. However, they might impede the lithium anode from being stable. In comparison, Metal-based materials are promising in favor of rapid 3D Li_2_S deposition due to their interfacial synergistic catalytic and electronic modulation effects.

### Shuttle Effect

The shuttle effect originating from the dissolution of LiPSs at the cathode–electrolyte interface leads to the loss of active materials and rapid capacity decay. Capturing LiPSs is the most popular method in the solid–liquid–solid stepwise reaction pathway among the explored strategies to lessen the severe shuttle effect of LSBs. Regulating the reaction pathway to limit the contact between the electrolyte and LiPSs is paid more and more attention. Herein, we will review the most recent advances in the perspective of diverse reaction patterns to mitigate the shuttle effect.

#### Adsorption of LiPSs in the Solid–Liquid–Solid Pathway

In the traditional solid–liquid–solid pathway, it is common to use diverse materials as the S host to absorb the LiPSs to prevent shuttling in various ways. To date, functional carbon materials, polar metal compounds, polymers, and MXenes have been all widely applied for interfacial modification in the hope of achieving physical/chemical adsorption of LiPSs at the cathode–interface interface.

(1) Functional carbon materials

**Fig. 9:**
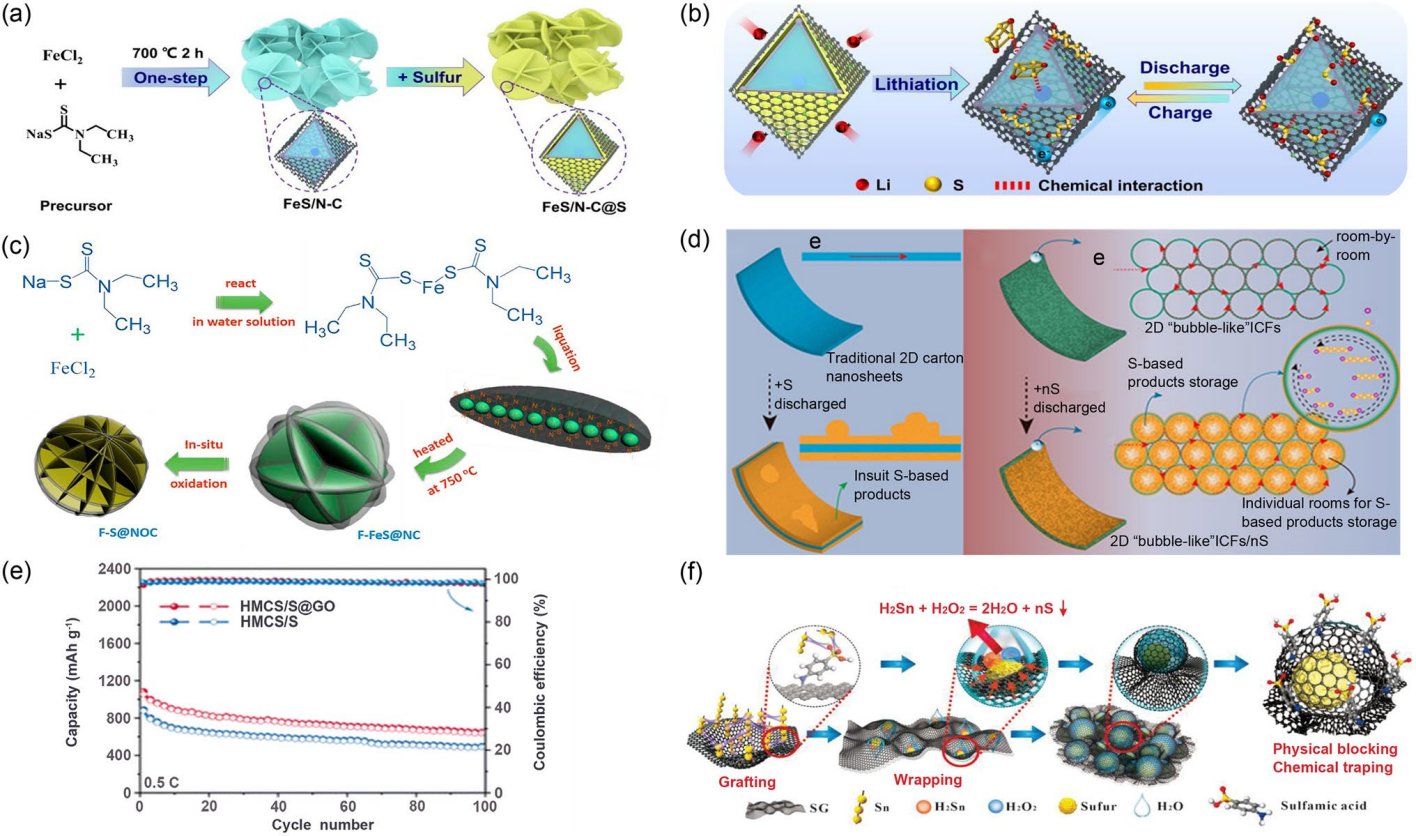
The schematic of **a** synthesis procedure and **b** strong interaction with LiPSs during the charge/discharge process of FeS/N–C@S nanocomposite cathode. **a**, **b** Reproduced with permission from Ref. [128], Copyright 2021, Elsevier. **c** Schematic preparation of F-S@NOC composite. Reproduced with permission from Ref. [130], Copyright 2018, Elsevier. **d** Schematic comparison between the traditional 2D carbon/S and bubble-like ICFs/nS cathodes. Reproduced with permission from Ref. [131], Copyright 2017, American Chemical Society. **e** Cycling performance comparison between the HMCS/S composite and HMCS/S@GO cathodes. Reproduced with permission from Ref. [132], Copyright 2022, Royal Society of Chemistry. **f** Self-caging mechanism for the growth of yolk-shell graphene@S particles. Reproduced with permission from Ref. [133], Copyright 2021, John Wiley and Sons

Functional carbon materials have gained extensive popularity in enhancing the energy storage performance of LSBs due to their unique physicochemical properties that benefit from distinct functional groups. Taking the N-doped carbon (NC) as an example, the N atoms are well known to be point defects and can significantly increase the carbon’s overall conductivity and polarity. This enhancement makes it possible to bond LiPSs and confine the shuttle effect at the cathode–cathode–electrolyte interface [126]. Additionally, the interconnected NC increases the electric contact at the interface and improves the performance with a promising initial discharge capacity [127]. When the NC is further composited with transition metal compounds such as FeS (Fig. 9a, b) [128] and CeO_2_ [129], the exposure of high active sites at the interface can be dramatically increased. This not only protects the cathode from depletion in electrolytes but also promotes redox reactions and enhances chemical adsorption of LiPSs through interaction with N atoms, thereby improving the overall conversion efficiency. Moreover, uniform depositing of the product Li_2_S at the cathode-side interface can be observed upon additional cycling, meaning a significant advance in desired capacity and long-term cycling stability. When carbon is co-doped with N and O, the surrounding electronic structure of O atoms can be greatly adjusted, strengthening the bond between S and O by enhancing the chemical interaction [126]. As shown in Fig. 9c, Wang et al. prepared a composite cathode of flower-like N/O co-doped carbon coated S (F-S@NOC). Benefiting from the exposure of more N/O functional polar groups, the cathode was able to anchor LiPSs, exhibiting excellent rate performance and cycling stability with a low decay rate of only 0.069% per cycle over 500 cycles at 1 C [130].

With sufficient oxygen-containing functional groups such as epoxy, carboxyl, and hydroxyl groups, graphene oxide (GO) is considered a highly effective polar material for adsorbing LiPSs. Wu et al. used GO wrapped interconnected carbon fabrics/S (ICFS) as the cathode and successfully sealed the open access of ICFs to anchor S (Fig. 9d) [131]. Thanks to the polar adsorption of GO and the enhanced conversion kinetics by electronically uneven polarized S, the specific energy capacity of the pouch cell reached up to 1.55 Ah@315.98 Wh Kg^−1^ at 0.1 C. However, GO fragments are prone to accumulate, which leads to a significant exposure decrease in active surface and a deterioration of performance. Therefore, 3D GO with straight mesoporous access is designed to wrap on the surface of hollow carbon spheres (HMCS@GO) by electrostatic adsorption and interfacial van der Waals interactions [132]. It anchors LiPSs effectively as the S host. When the hollow carbon shell and mesopores that facilitate the Li^+^ diffusion is combined, the conversion of LiPSs is promoted. Therefore, the initial discharge capacity of 1054 mAh g^−1^ is delivered at 0.5 C with a capacity retention rate of 60.2% after 100 cycles (Fig. 9e). Sulfonated graphene (SG), as another functional carbon material, was applied by Yu et al. to encapsulate S particles inside the atomic shells, forming self-assembled nanocages that can polarize S_8_ through sulfonate groups on its surface (Fig. 9f) [133]. SG and S_8_ interact strongly during the reaction due to the strong electronic absorption. Therefore, the excellent nanocage stability, the tight wrapping on S, and the superior chemisorption of the sulfonic groups on LiPSs together construct a stable cathode interface and realize the advanced electrochemical performance of LSBs.

Although functional carbon materials possess a distinct ability to anchor LiPSs and excellent ionic/electronic conductivity to accelerate the reaction kinetics on a large scale, combining desired functionality to increase energy density remains a challenge that requires comprehensive consideration of the physical and chemical properties of various components.

(2) Polymer-based materials

Polymer-based materials have good affinity to LiPSs due to their rich polar functional groups (C–N, C–S, C–O) and conjugated structures with alternating C–C and C=C bonds [134]. O, N, and S heteroatoms in polar functional groups can achieve considerable chemisorption to LiPSs. In addition, some of them pose excellent electric/ionic conductivity, such as polypyrrole (PPy) [134–136], polyaniline (PANI) [137], and poly(3,4-ethylene dioxythiophene) (PEDOT) [138]. The introduction of polymer-based materials will also reduce the dissolvation of the LiPSs at the cathode–electrolyte interface.

**Fig. 10:**
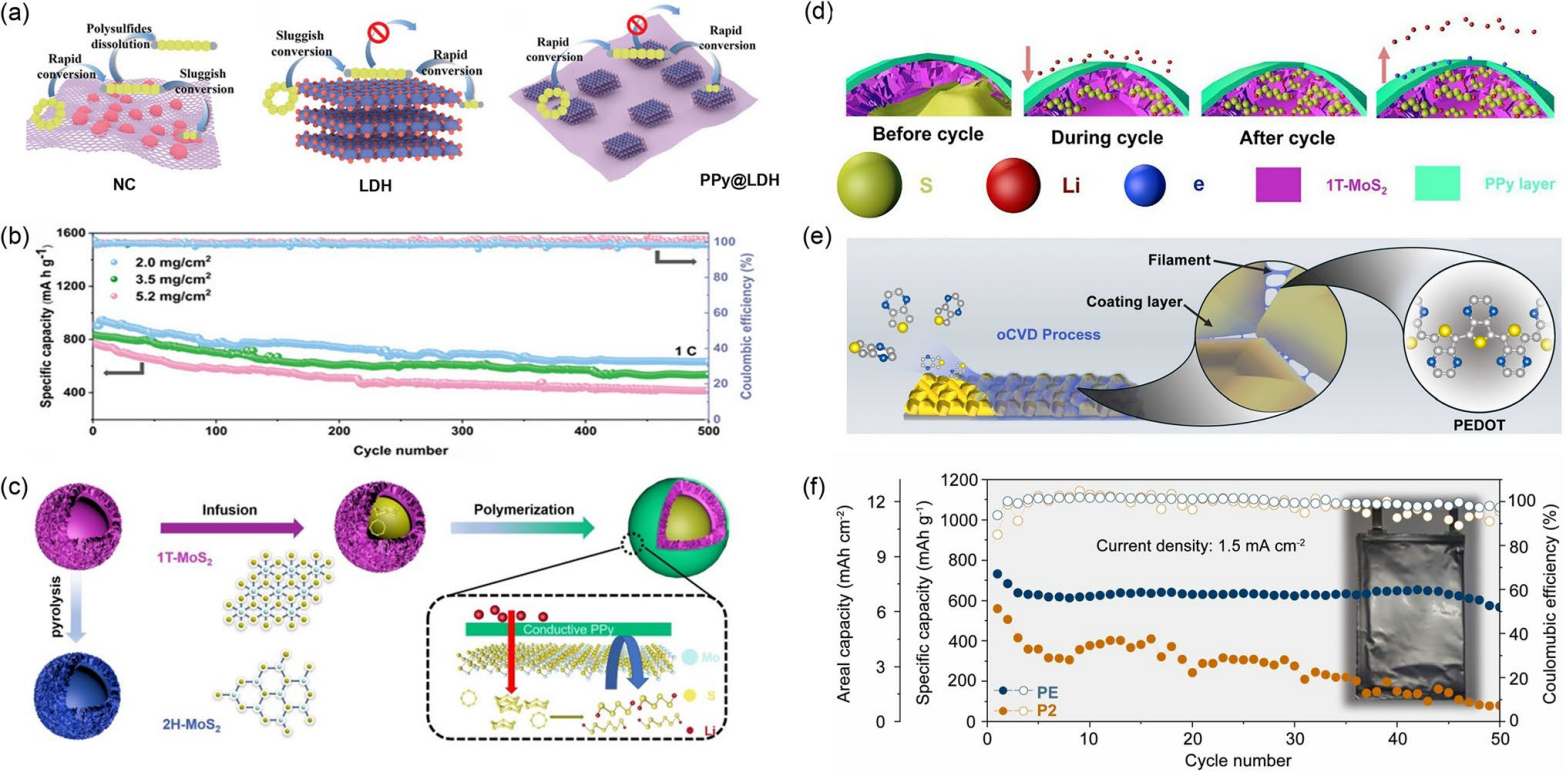
**a** Schematic illustrations of S adsorption and catalyzation on NC, LDH, and PPy@LDH. **b** Long-term cycling performance of the PPy@LDH-S cathodes with different S loadings in cells. **a**, **b** Reproduced with permission from Ref. [141], Copyright 2023, John Wiley and Sons. **c** Schematic illustrations of synthesis and **d** LiPSs trapping mechanism of 1 T-MoS_2_-S@PPy cathode. **c**, **d** Reproduced with permission from Ref. [135], Copyright 2022, Elsevier. **e** The fabrication process of o-PEDOT modified S cathode. **f** Cycling performances of Li–S pouch cells with the P2 and PE cathodes, respectively. **e**, **f** Reproduced with permission from Ref. [145], Copyright 2023, Elsevier

PPy exhibits an electronic conductivity of 2–100 S cm^−1^ due to its hydrophilic and interconnected five-membered pyrrole rings. The electronic feature and long-chain structure allow its strong interactions with LiPSs and promote the redox reaction of LSBs [139]. Geng et al. used a ~ 55-nm-thick PPy layer to cover the hollow metal–organic framework (MOF) [140], which showed a significantly improved electrochemical rate and cycling performance. Dong et al. also applied PPy to coat the hollow layered double Ni-Co hydroxide (Ni-Co LDH) (Fig. 10a) [141], not only enhancing the chemisorption of LiPSs and overall electronic conductivity but also increasing the surface reaction rate and electrocatalytic activity of the Ni-Co LDH, facilitating reversible conversion between LiPSs and Li_2_S_2_ [142, 143]. At a current density of 1 C, the initial discharge capacity was 907.2 mAh g^−1^ and maintained at 633.4 mAh g^−1^ after 500 cycles (Fig. 10b). Coupling conductive polymers with polar materials can directly avoid the LiPSs to contact with electrolyte. For example, both highly defective (amorphous) black-TiO_2_ [136] and hollow 1 T-MoS_2_ skeleton (Fig. 10c, d) [135] can catalyze the conversion and limit the LiPSs dissolution through a synergistic interaction with the PPy layer. Poly(3,4-ethylene dioxythiophene): poly(styrenesulfonate) (PEDOT:PSS) is also designed to form an interface layer on the spherical double-layered hollow C/S composite. This unique design enabled multiple components in the cathode to work well and showed outstanding cycling stability with a capacity decay rate of 0.097% during 500 cycles [144].

Polymer-based materials can also be used to modify the cathode–electrolyte interface on a cathode scale. Oxidative chemical vapor deposition (oCVD) is an emerging deposition process that generates conjugated polymer films by gas-phase reaction with high conductivity, excellent homogeneity, and conformal properties [146]. Zhang et al. deposited o-PEDOT on cathode using oCVD, which was able to fix the LiPSs through physical encapsulation and chemisorption, effectively limiting the dissolution of LiPSs in the electrolyte (Fig. 10e) [145]. Meanwhile, the o-PEDOT layer with a highly conductive network structure can provide more charge and mass transfer sites for insoluble LiPSs, facilitating the solid–solid conversion reaction kinetics. The S cathode with o-PEDOT in pouch cell provided an initial capacity of 732.8 mAh g^−1^ at the current density of 1.5 mA cm^−2^ with a high S loading of 11 mg cm^−2^. It has a high discharge capacity of 567.1 mAh g^−1^ even after 50 cycles (Fig. 10f).

Polymers have been demonstrated to relieve the shuttle effect for enhancing the electrochemical performance of LSBs, but further efforts are needed to explore scalable and economically advantageous technologies. Meanwhile, controlling coating thickness to create a uniform cathode–electrolyte interface that maximizes performance is also a future task.

(3) Polar metal-based compounds

Generally, polar–polar interactions are stronger than polar–nonpolar interactions, and binding energy is one of the key factors in evaluating the dominance of adsorption ability [147]. Since LiPSs are polar compounds, the introduction of polar metal-based compounds can also adsorb the LiPSs through polar–polar interactions.

**Fig. 11:**
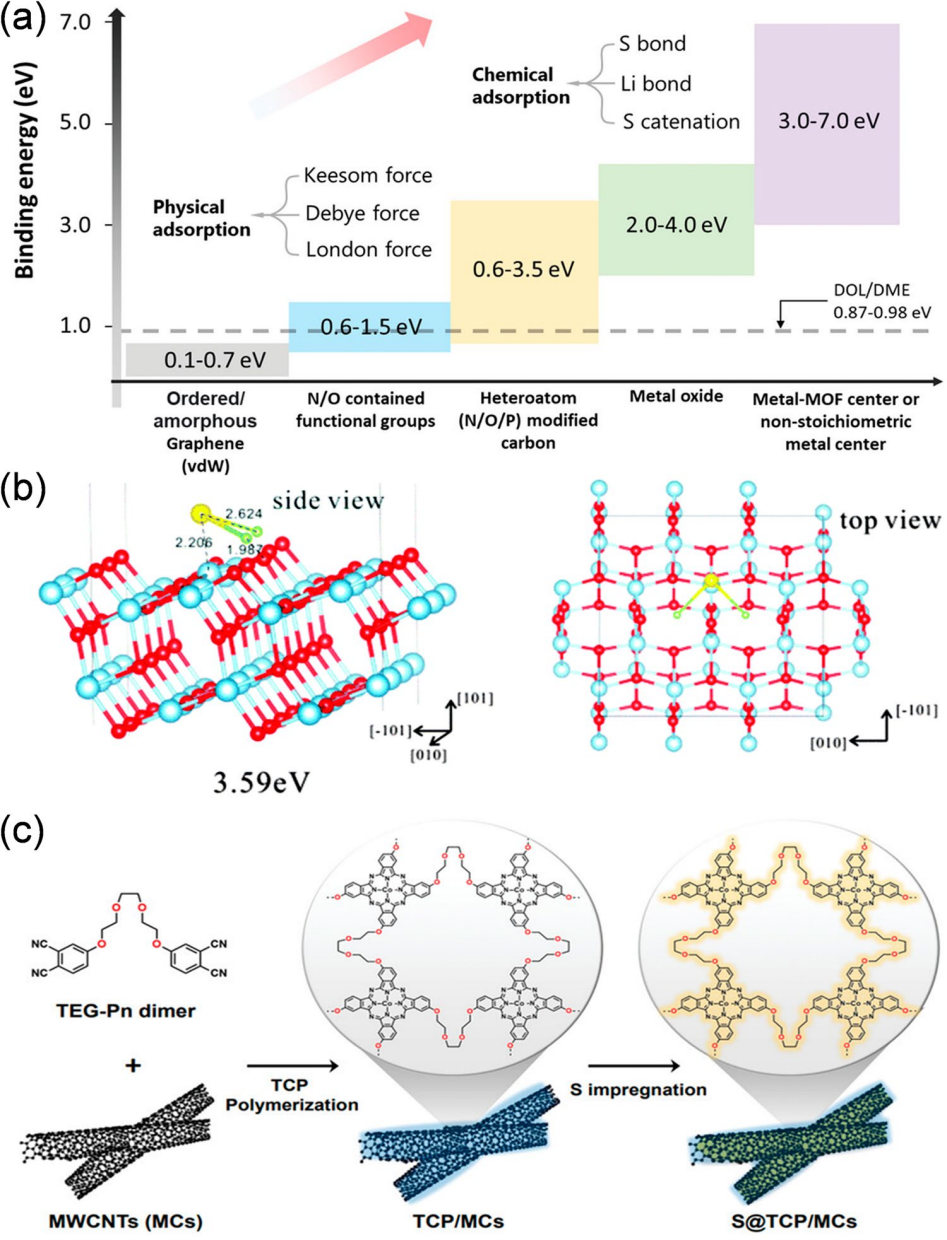
**a** Binding energy scope of categorized adsorbents to LiPSs. Reproduced with permission from Ref. [148], Copyright 2022, John Wiley and Sons. **b** Adsorption configuration of Li_2_S on anatase-TiO_2_ (101) surface. Reproduced with permission from Ref. [154], Copyright 2016, Royal Society of Chemistry. **c** Schematic preparation of the S@TCP/MCs electrode. Reproduced with permission from Ref. [155], Copyright 2023, John Wiley and Sons

As shown in Fig. 11a, the binding energy for chemical adsorptions of the polar metal compounds (such as metal oxides and metal MOF center or non-stoichiometric metal center) is much higher than that of the normal physical adsorptions [148]. To enhance the reaction performance of cathode–electrolyte interface, polar metal compounds with rich polar active sites have captured wide attention in LSBs research. They exhibit more efficient adsorption to LiPSs via not only physical trapping but also through strong chemical bonds, such as the Li bond or S bond [149, 150]. With fruitful works completed, a variety of polar metal compounds, including oxides, sulfides, and nitrides, have been widely proposed as appropriate interface modification materials in LSBs [150]. Moreover, some transition metal oxides (WO_3_ [151], CeO_2_ [152], and MoS_2_ [153]) show good catalytic properties and accelerated redox kinetics of the S conversion.

Yu et al. reported an effective way to alleviate the shuttling of LiPSs by coating TiO_2_ on the NG/S composite cathode [154]. Due to TiO_2_ acting as a Lewis acid and LiPSs acting as a Lewis base, electrons are transferred to NG/S to form Li-N and S-Ti bonds with LiPSs during the reaction. Therefore, the TiO_2_-NG/S cathode maintains a high discharge capacity of 918.3 mAh g^−1^ after 500 cycles due to the strong binding energy of 3.59 eV (Fig. 11b). Kim et al. designed a novel polymeric cobalt (Pc) containing triethylene glycol linkers (TCP) to coat on multiple wall carbon nanotubes (MCs) through a strong *π*–*π* interaction and formed a polar TCP/MCs composite (Fig. 11c) [155]. The TCP creates a lipophilic environment for the uniform distribution of Li^+^ active sites, thus accelerating Li^+^ diffusion [156, 157]. The lithophilic triethylene glycol (TEG) could anchor Li^+^ to the active site of LiPSs, and the Co atom will accept electrons from S atoms in Li_2_S_6_, subsequently forming a stable Co-S bond. Meanwhile, the N atoms in TCP and TEG linkers can form N-Li and O-Li bonds with the Li atoms in Li_2_S_6_, and the TCP layer also provides various Lewis acid–base binding sites for LiPSs, preventing the formation of insulative S composite species.

Both Yu et al. and Kim et al. developed different approaches to enhance the conversion of LiPSs by creating more favorable active sites for adsorption. It is worth noting that stronger binding energy is not always beneficial. When it exceeds 5 eV, LiPSs may be trapped at the cathode surface, hindering its conversion and causing secondary dissolution [158]. Polar metal compounds with moderate adsorption ability are preferred in LSBs. However, most metal oxides have poor electronic conductivity, leading to slow redox kinetics and inevitably impeding the direct LiPSs conversion at the interface [159].

(4) MXenes

As a class of 2D materials with polar characteristics, thermal stability, and manipulable Lewis acidic surface, MXenes are extensively used to modify the cathodes to confine the LiPSs shuttling of LSBs [160]. Compared to most polar metal compounds, MXenes tend to have excellent conductivity to accelerate the interface reaction kinetics [161].

**Fig. 12:**
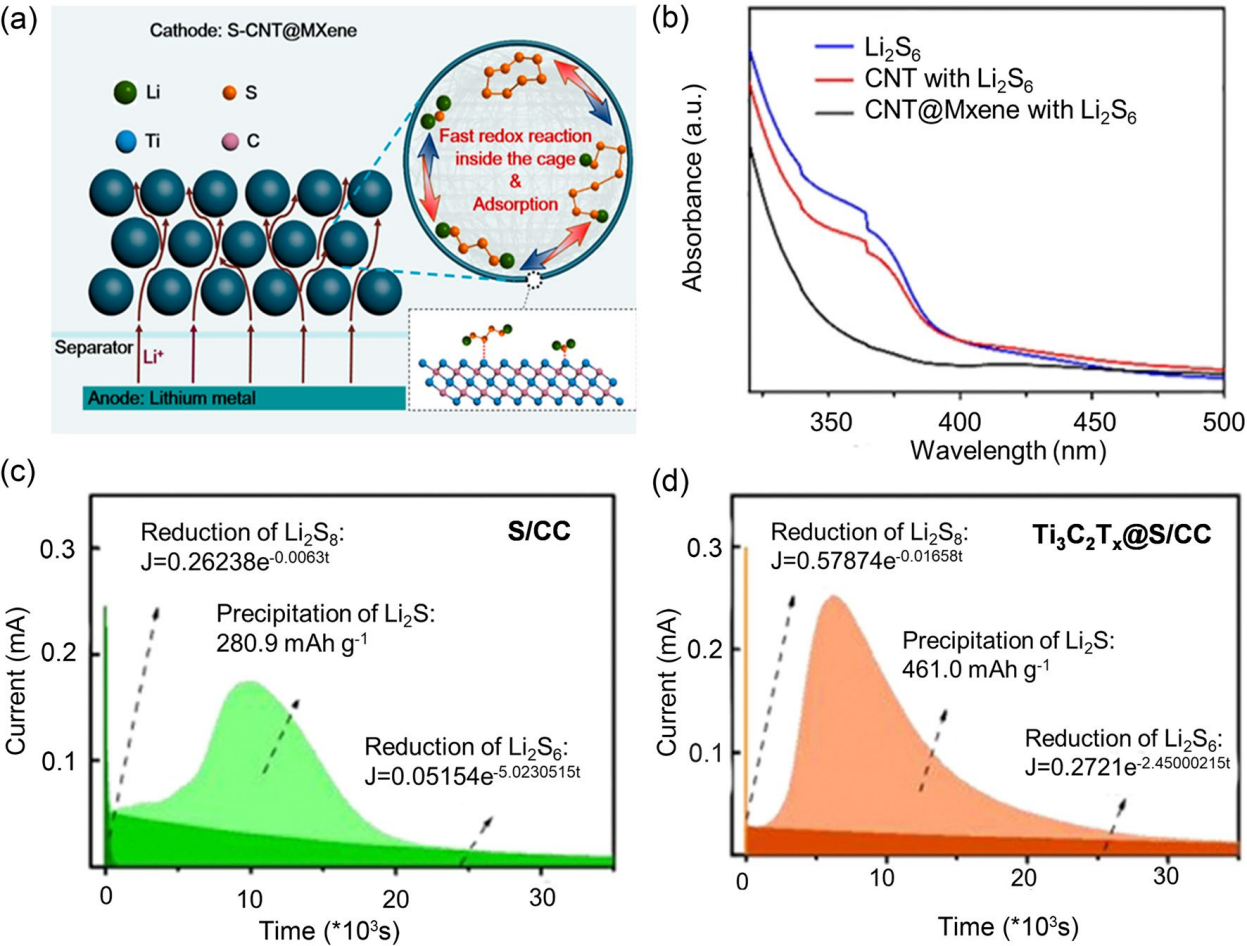
**a** Schematic illustration of the synthesis of 3D S-CNT@MXene cage spheres. **b** UV/vis spectra of the Li_2_S_6_, CNT with Li_2_S_6_ and CNT@MXene with Li_2_S_6_. **a**, **b** Reproduced with permission from Ref. [162], Copyright 2021, Elsevier. Reduction of Li_2_S_8_/Li_2_S_6_ and precipitation of Li_2_S during potentiostatic discharge of the Li_2_S_8_/tetraglyme catholyte on **c** CC and **d** Ti_3_C_2_T_x_@CC at 2.05 V. **c**, **d** Reproduced with permission from Ref. [163], Copyright 2021, Elsevier

In a study by Wang et al. (Fig. 12a), three-dimensional S-CNT@MXene cages were reported, where ultrathin MXene nanosheets were utilized around S-CNT porous spheres [162]. The spherical structure avoids the re-stacking of MXene and makes full use of its active sites, improving the Li^+^ diffusion at the interface. Due to the abundant terminal groups of -OH, -O, and -F on the MXene surface, the composite exhibits significant chemical adsorption with LiPSs and facilitates electrolyte penetration. The UV/Vis spectra showing a remarkable blueshift of the absorption edge further confirmed the strong chemical interaction between CNT@MXene and LiPSs (Fig. 12b). Yin et al. synthesized S-impregnated carbon cloth cathode covered with Ti_3_C_2_T_x_ flakes (Ti_3_C_2_T_x_@S/CC) [163]. The Ti_3_C_2_T_x_ layer not only physically confines the LiPSs but also chemically interacts with them due to its hydroxyl group to form surface thiosulfate species, which traps LiPSs and converts them directly into Li_2_S through disproportionation. Also, the exposed acid Ti sites on the Ti_3_C_2_T_x_ can strongly adsorb LiPSs through the formation of Ti-S bonds, further promoting the direct nucleation of Li_2_S. Consequently, the Li_2_S precipitation capacity of Ti_3_C_2_T_x_@CC (461.0 mAh g^−1^) (Fig. 12c) is higher than that of CC (280.9 mAh g^−1^) (Fig. 12d).

Despite the strong anchoring ability of MXene to LSBs at the cathode–electrolyte interface, it is susceptible to oxidation in water and air due to the exposed metal atoms, leading to changes in properties [164, 165]. Therefore, improving its stability is an urgent problem that needs to be solved.

In the conventional solid–liquid–solid three-phase conversion mechanism, the troublesome shuttle effect begins at the liquid-phase transition stage. Therefore, the common ground these tactics have is they all aim to relieve the influence of the dissolution of the soluble LiPSs at the cathode–electrolyte interface and have made remarkable progress in the laboratory condition. However, they could only limit the shuttling of LiPSs but could not eliminate the liquid-phase transition.

#### Transformation from Solid–Liquid–Solid Pathway to Solid–Solid Pathway

In contrast to the solid–liquid–solid reaction mechanism, which involves anchoring the LiPSs through physical or chemical operations to suppress the shuttle effect as much as possible, the transition from a solid–liquid–solid pathway to a solid–solid pathway is anticipated to eliminate the shuttle effect. The process of achieving this transition is that a small amount of LiPSs is produced during the initial reduction stage, and subsequently the electrolyte reacts swiftly with LiPSs or undergoes in situ polymerization to form a dense CEI layer. As a result, a complete physical isolation of S from the electrolyte is achieved, and the redox pathway of S is transformed from the solvation–deposition mechanism to a solid-phase mechanism. In the mechanism, it becomes evident that the liquid–solid–solid reaction predominantly generates a dense CEI layer through electrolyte modification.

**Fig. 13:**
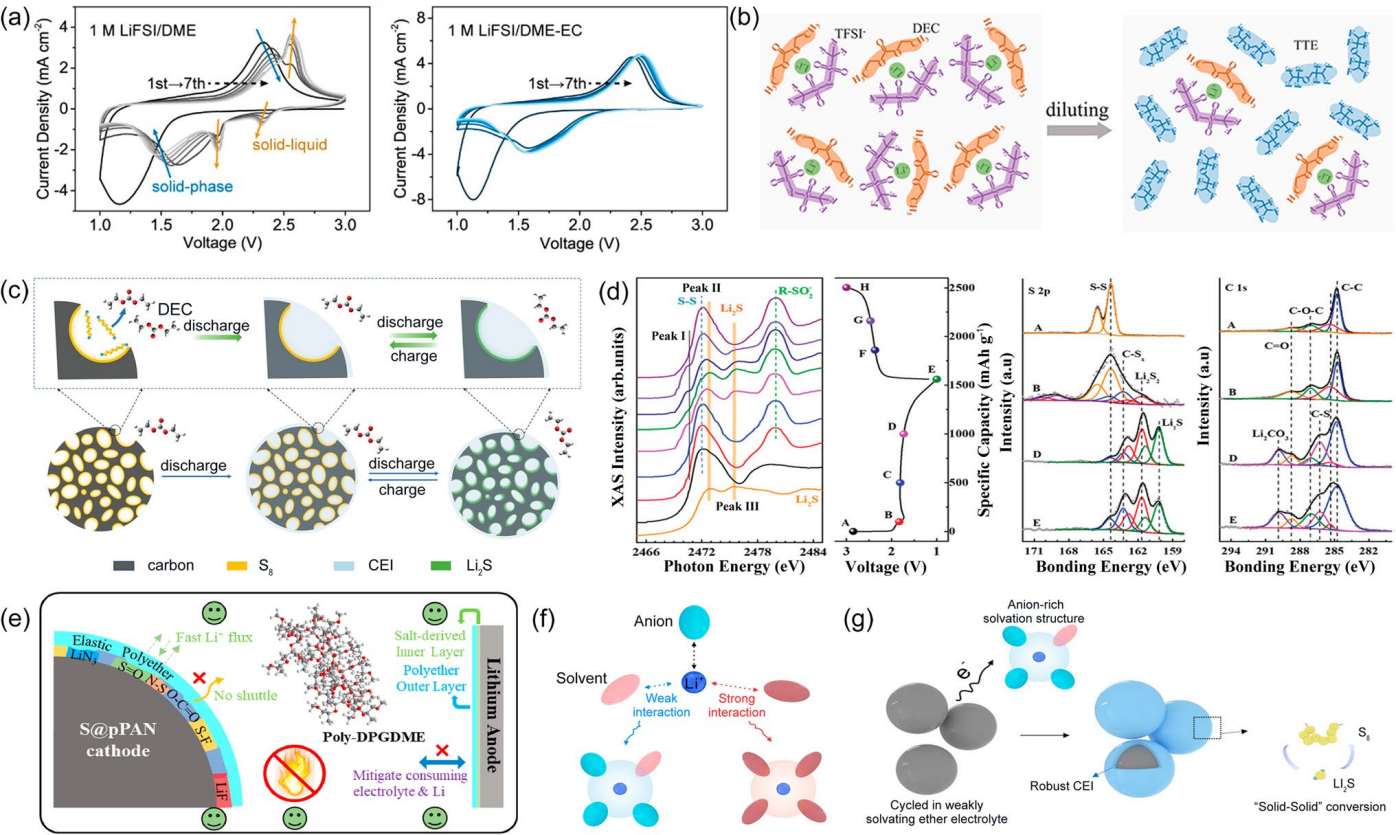
**a** CV curves of the Li-SPAN half cells in 1 M LiFSI/DME and 1 M LiFSI/DME-EC electrolytes, respectively. Reproduced with permission from Ref. [114], Copyright 2021, American Chemical Society. **b** Solvate structure illustrations of the HCE (left) of LiTFSI + DEC and the LHCE (right) of LiTFSI + DEC + TTE, in which the LiTFSI, DEC, and TTE are used as examples for salt, solvent, and diluent, respectively. **c** Schematic illustration of the in situ formation of the CEI layer on the KB/S surfaces in the localized high-concentration carbonate-based electrolyte. **d** S K-edge spectra of KB/S electrodes in the carbonate LHCE at given discharge/charge steps and the corresponding GCD curve of the cell; XPS spectra of the electrode at given discharge states: S 2*p*, C 1*s*. **b**-**d** Reproduced with permission from Ref. [166], Copyright 2021, John Wiley and Sons. **e** The superiorities of the proposed DPGDME electrolyte toward both electrodes. Reproduced with permission from Ref. [169], Copyright 2022, Elsevier. **f** Illustration of Li^+^-solvent interactions. **g** Conversion mechanism of SPAN in weakly solvating ether electrolyte. **f**, **g** Reproduced with permission from Ref. [170], Copyright 2023, John Wiley and Sons

Carbonates, such as EC [114], diethyl carbonate (DEC) [166], and VC [167], can interact with LiPSs by nucleophilic reaction, resulting in the formation of polycarbonate organic precipitates with strong capabilities to impede the dissolution of LiPSs. The CEI layer composed of polycarbonate organic substances deposited on the cathode surface can achieve good physical isolation between the cathode and electrolyte, allowing only Li^+^ to pass through and access the interior of cathode. Subsequently, the S conversion mechanism transitions into a solid–solid route. Reflected in the cyclic voltammetry (CV) curves, the redox peaks corresponding to the solid–liquid reaction gradually disappear and only solid-phase reaction maintains by following the formation of CEI layer, indicating complete inhibition of the LiPSs generation, as depicted in Fig. 13a [114]. Due to the nucleophilic reaction between carbonate compounds and LiPSs, this unique solid-phase transformation mechanism demonstrates high reversible cycle performance. A LSB utilizing S/C_FS_ as a cathode and VC acts as a co-solvent and exhibits outstanding charge/discharge characteristics with an original capacity of 1557 mAh g^−1^ and a peak cycling efficiency of 99.9% over 500 cycles [167].

Localized high-concentration carbonate electrolyte (LHCE) represents another way to harness carbonate solvents which are acquired by adding an inert diluent to the high-concentration electrolytes (HCEs). The reduced viscosity and improved ionic conductivity resulting from inert diluents can maintain the advantages of HCEs in forming a dense CEI layer [168]. For example, He et al. added the 1,1,2,2-tetrafluoroethyl-2,2,3,3-tetrafluoropropyl ether (TTE) to a DEC/FEC + LiTFSI system and created a LHCE (Fig. 13b) [166]. The addition of TTE does not coordinate with Li^+^ but the electrolyte dilution promotes Li^+^ migration. During the early stage of discharge, the nucleophilic reaction between carbonate and LiPSs gives rise to a CEI layer that exclusively allows only Li^+^ to pass through, and S and Li^+^ are directly converted to Li_2_S/Li_2_S_2_ without further generation of LiPSs (Fig. 13c). As shown in Fig. 13d, the analysis of the S-k edge spectra and XPS spectra reveals that KB/S cathode undergoes a solid-phase conversion with the formation of a CEI layer comprising insoluble inorganics (LiCO_3_ and LiF) and thiocarbonates on the electrode surface. However, it should be noted that the excessive diluent may decrease the proportion of carbonate, altering the solvent structure and impacting ionic transport, thus affecting the availability of active material.

In conventional dilute ether electrolytes, the S transition entails a solid–liquid–solid pathway involving the generation and dissolution of LiPSs, resulting in obvious shuttle effect. However, ether electrolytes with low solubility toward LiPSs are beneficial for maintaining stable interfacial phases. Chen et al. utilized a non-toxic and non-flammable dipropylene glycol dimethyl ether (DPGDME) as the solvent [169]. DPGDME can be in situ electrochemically polymerized during cycling and deepened with the cycling continuance, resulting in abundant polyethers on the cathode surface that builds an elastic CEI layer with low impedance (Fig. 13e) for fast Li^+^ diffusion. The low LiPSs solubility of the polymer effectively maintains the solid–solid conversion, ensuring high capacity (1645.3 mAh g^−1^ based on S), excellent cycling stability (99.5% retention over 400 cycles), and ultra-high average coulombic efficiency (CE) over 99.9995%. Ma et al. proposed low-cost and low-density weakly solvated electrolytes based on butyl methyl ether (BME) with low solvation power [170]. Compared with common ether solvents, the single ether O-bond of BME results in a lower electron donation, which reduces the coordination capacity of BME and the solubility of LiPSs in BME solvents (Fig. 13f). The lower coordination ability causes the solvation shell of the electrolyte to be dominated by FSI^−^, which together with LiPSs forms stable CEI layer, effectively preventing LiPSs from dissolving and shuttling to the electrolyte, thereby realizing the solid–solid conversion (Fig. 13g).

Although a solid–solid pathway can be modulated with an organic-dominated CEI layer and exhibits excellent sealing to the cathode, the rate performance of the LSBs is relatively poor compared with dissolved-deposition mechanism. To improve the overall performance in the future, therefore, it may be considered to construct an organic–inorganic hybridized CEI layer, of which the crucial part is accurately controlling the ratio of organic and inorganic components. The success of new ether-based electrolyte demonstrates that regulating the solvation structure through proper selection of salts and solvents is an acceptable means to establish a hard-closed CEI layer to realize the solid–solid conversion.

#### Avoiding LiPSs Generation Through All-Solid–Solid Reaction

Compared with the above-mentioned reaction transition from solid–liquid–solid to solid–solid, the all-solid–solid conversion mechanism exclusively refers to the solid-phase conversion of S without the formation of any soluble LiPSs and can completely eliminate the shuttle effect. There are two main routes to achieve this. One is to directly sever the contact between S and electrolyte by establishing spatial restrictions at the cathode–electrolyte interface before cycling, while the other entails forming S-containing composites as the active substance through covalent bonding.

**Fig. 14:**
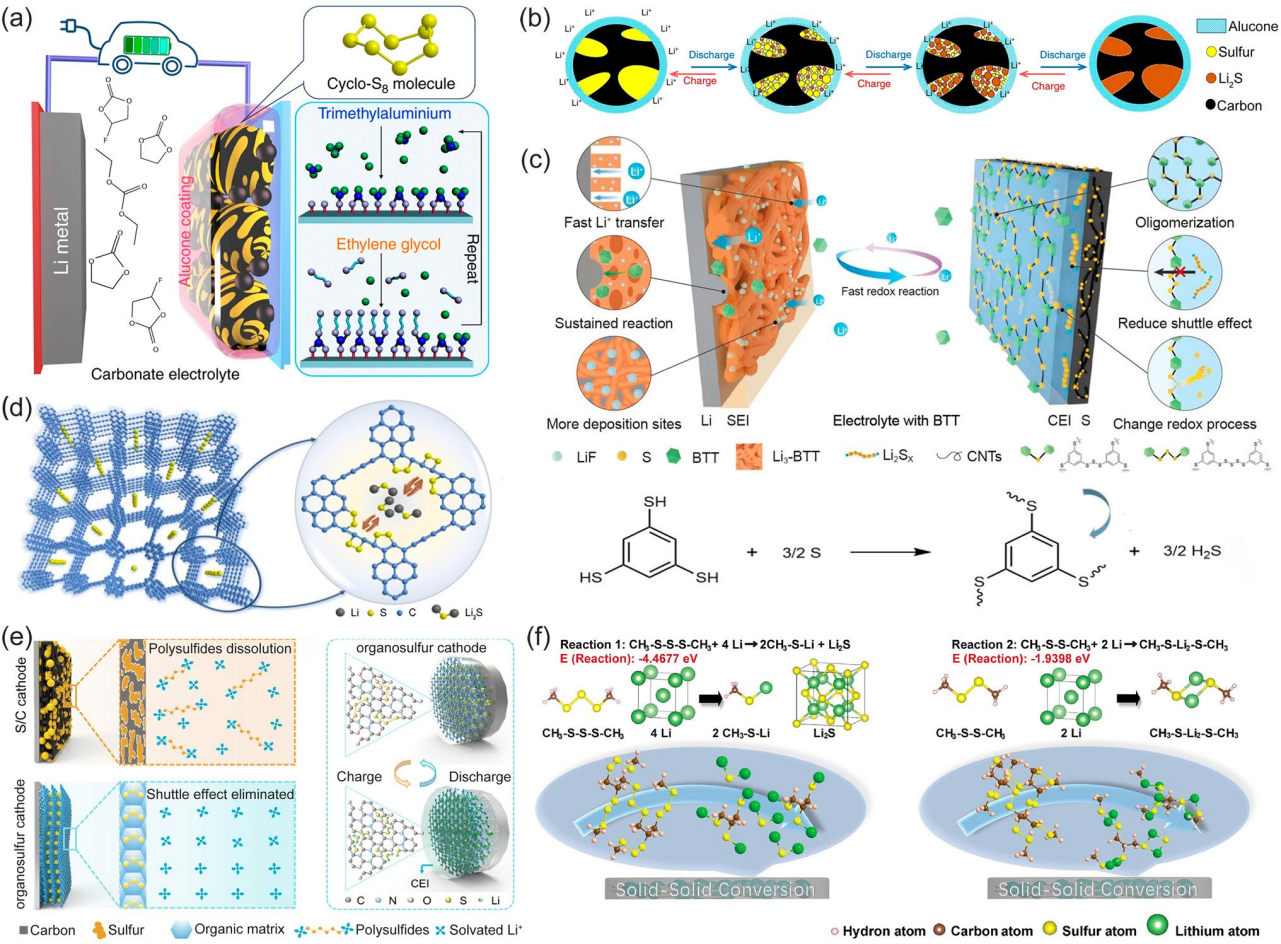
**a** Schematic diagram of alucone coating on carbon/S (ring-S_8_) cathode and **b** proposed reaction mechanism in the carbonate electrolyte. **a**, **b** Reproduced with permission from Ref. [171], Copyright 2018, Springer Nature. **c** D-SEIs are formed on the interfaces of the anode and cathode. Reproduced with permission from Ref. [172], Copyright 2021, Springer Nature. **d** Schematic diagram of the SGPOF with a short S-chain. Reproduced with permission from Ref. [173], Copyright 2021, American Chemical Society. **e** Schematic structures of S/C cathode following solid–liquid–solid reaction with LiPSs dissolution (above) and the organosulfur cathode (below) following solid–solid reaction with eliminated shuttle effect, as well as the structural reorganization of organosulfur cathode during the reaction process. Reproduced with permission from Ref. [174], Copyright 2023, Elsevier. **f** Calculation of energy changes of possible lithiation reactions, bond length, and reaction formula for CH_3_–S–S–S–CH_3_ (Reaction 1) and CH_3_–S–S–CH_3_ (Reaction 2), and their proposed electrochemical conversions. Reproduced with permission from Ref. [175], Copyright 2022, Elsevier

Employing a suitable layer represents an effective means of creating a physical separation at the cathode-interface. Owing to the precise control over the thickness and chemical composition on a molecular scale, molecular layer deposition (MLD) enables the creation of an effective separation layer on the cathode surface. For instance, Li et al. deposited a Li^+^ accessible alucone film on the surface of C/S cathode via MLD [171]. The alucone film effectively achieved physical isolation between the cathode and carbonate electrolyte, forming a stable interface and preventing side reactions (Fig. 14a). During cycling, the cyclo-S_8_ in the conductive carbon matrix was directly converted to Li_2_S through a solid-phase redox reaction rather than the complex solid–liquid–solid conversion (Fig. 14b). Another route involves electrolyte modulation for in situ CEI formation. Guo et al. developed an electrolyte additive of 1,3,5-benzenetrithiol (BTT), which combined with S alone or in pairs to react with Li^+^, producing Li_2_S_x_ on the cathode surface and lithium benzene trithiol (Li_3_-BTT) on the anode surface, respectively (Fig. 14c) [172]. This interfacial reaction differs from the usual in situ reaction independent of LiPSs generated in the early cycles, instead of the in situ direct oligomerization of BTT and S forming a chemically and mechanically stable solid CEI layer. Based on this unique redox pathway fundamentally inhibiting the LiPSs production, the first discharge capacity is 1036 mAh g^−1^ at 1 C and maintains 907 mAh g^−1^ even after 300 cycles with 87.6% capacity retention.

Yi et al. thermally synthesized a graphdiyne-like porous organic framework (GPOF), of which highly active acetylene bonds reacted with S within the micropores at high temperature, forming a sulfide compound of SGPOF with C–S–S–C chains [173]. The short chains chemically react with the unsaturated carbon atoms of the GPOF through covalent bonding (Fig. 14d), resulting in only the solid-phase transformations among the low-molecular-weight sulfides and thereby eliminating the LiPSs generation at the cathode–electrolyte interface. When S is covalently immobilized on the triallyl isocyanurate to synthesize S-triallyl isocyanurate organosulfur polymer composites (STIs) that are used as actives, the triallyl monomer and S will form a cyclic structure embedded with short polysulfur chain, avoiding detrimental transitions of the long-chain LiPSs in the discharge/charging process (Fig. 14e) [174]. Under high S loading (4.5 mg cm^−2^) and low S electrolyte (8 uL mg^–1^) conditions, the pouch cell showed almost no capacity degradation over 125 cycles. Zhang et al. prepared polymers with a high S content containing disulfide chains (DSP) and trisulfide chains (TSP) as novel active materials for LSBs [175]. As shown in Fig. 14f, DFT calculations showed that the DSP and the TSP have different lithiation products and reaction pathways from the monolithic S, avoiding the generation of LiPSs during the conversion process and providing a stable cathode–electrolyte interface. The solid–solid conversion mechanism can be realized through spatial confinement but requires the preparation process as a pressing subject. In contrast, S-containing composites are easier to commercialize and it is feasible to explore composites with higher S content.

## Summaries and Prospects

**Fig. 15:**
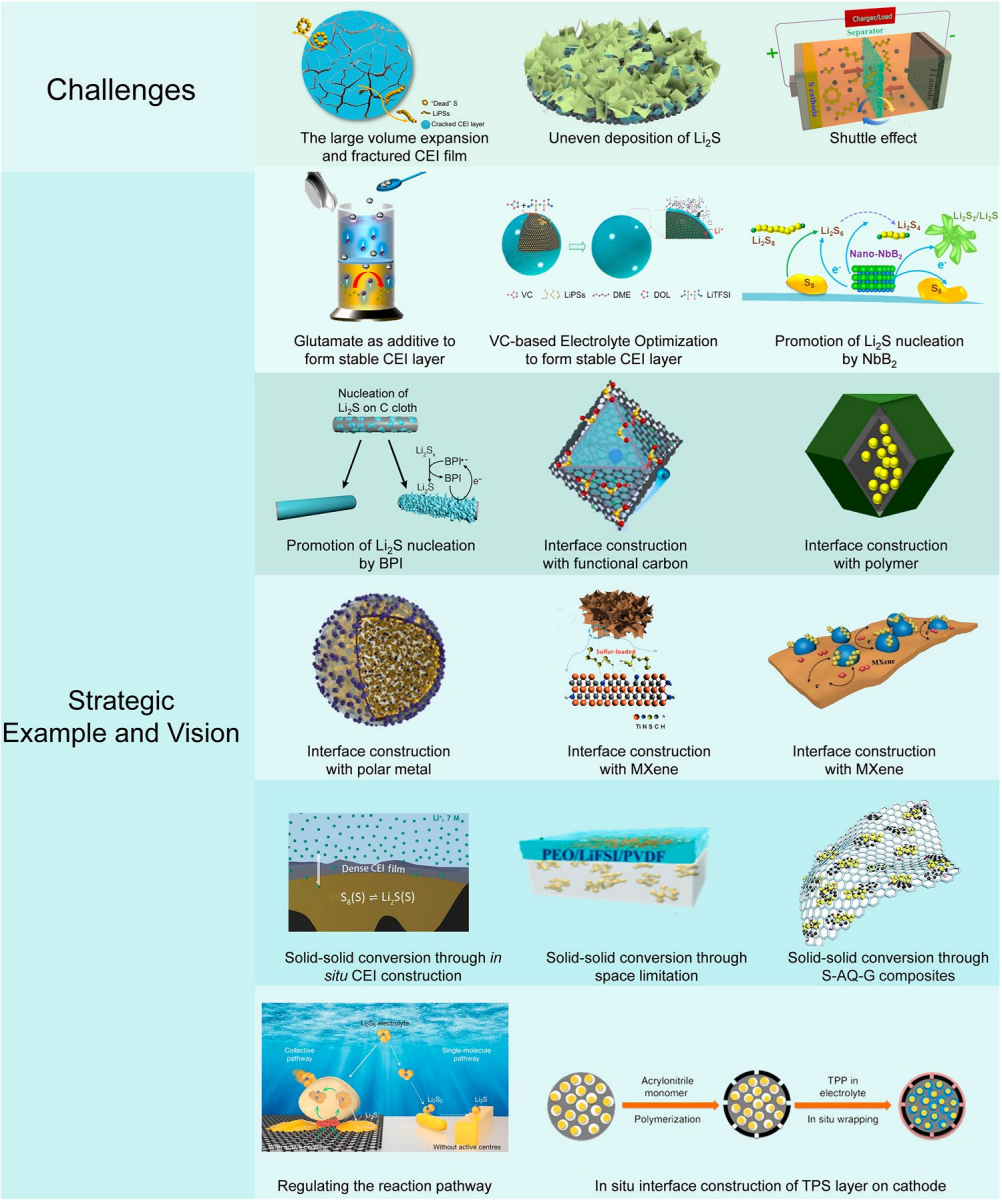
The challenges, strategic examples, and novel visions of the electrode–electrolyte interface of LSBs. Reproduced with permission from Ref. [177], Copyright 2023, Elsevier; Ref. [178], Copyright 2021, Elsevier; Ref. [179], Copyright 2019, American Chemical Society; Ref. [115], Copyright 2023, John Wiley and Sons; Ref. [180], Copyright 2022, American Chemical Society; Ref. [181], Copyright 2016, American Chemical Society; Ref. [128], Copyright 2021, Springer Nature; Ref. [182], Copyright 2023, MDPI; Ref. [183], Copyright 2020, Springer Nature; Ref. [184], Copyright 2018, Springer Nature; Ref. [160], Copyright 2019, Springer Nature; Ref. [185], Copyright 2023, Springer Nature; Ref. [186], Copyright 2023, Springer Nature; Ref. [187], Copyright 2018, Springer Nature; Ref. [176], Copyright 2019, Springer Nature; and Ref. [188], Copyright 2019, Springer Nature

LSBs with high specific capacity and low cost are viewed as one of the most promising candidates for the post-LIBs era. However, there are still a lot of cathode–electrolyte interface issues, such as shuttle effect and the structural changes, momentarily left in suspense impeding their practical application. To tackle the obstacles, manipulating the interface is gradually paid more and more attention. In this review, a thorough and systematic understanding of cathode–electrolyte interface issues and the corresponding state-of-the-art strategies are presented (Fig. 15) and well discussed. The strategies are classified according to the perspectives of structural enhancement and reaction mechanisms.

The CEI layer and deposition pattern of Li_2_S are directly connected to the stability of the cathode–electrolyte interface. Their optimal method also has a resemblance to adding electrolyte additives. It is worth mentioning that adding proper electrolyte additives could promote the formation of a CEI layer. The mechanism of how the CEI layer responds to certain additives should be further expanded in the future to direct the rational design of electrolyte additives. Either increasing the solubility or regulating the deposition pattern of Li_2_S proves favorable and effective. The high DN solvents can serve as the electrolyte to not only increase the solubility of Li_2_S but also stabilize the short-chain LiPSs to provide a novel deposition pathway. In particular, metal-based materials can promote the 3D deposition of Li_2_S while avoiding corrosion of the lithium metal anode that occurs in high DN electrolyte systems. However, the 3D deposition of Li_2_S on metal substrates comes at the expense of catalytic sites, so it needs further exploration on how to maintain the catalytic activity of the interface.

The dissolution of the LiPSs at the cathode–electrolyte interface that triggers the subsequent shuttle effect can lead to damage in both electrodes and capacity decline. In recent years, studies have shown that the traditional “solid–liquid–solid” mechanism can be modulated to inhibit the shuttle effect. The common strategy is adopting various materials to modify the cathode interface to restrain the LiPSs from shuttling. The option varies from functional carbon, polymers with rich polar functional groups, and polar metal compounds to MXenes. However, they could only mitigate the shuttle effect not eliminate it. The common ground these methods share is to avoid the dissolution of the LiPSs into the electrolyte, and some novel methods to regulate the reaction mechanism are under the same idea to avoid the liquid-phase transition at the cathode–electrolyte interface which can restrain the shuttle effect from the root. The transformation from the solid–liquid–solid pathway to the solid–solid pathway is achieved through the electrolyte modification, and the solid–solid reaction pathway can generally be modulated by either spatial restrictions between the cathode and electrolyte or the covalent bonding of the S-containing composites.

We believe that a full understanding of the cathode–electrolyte interface behavior is the key to improving the overall performance of the LSBs. Recently, it has been paid more and more attention. The insight into the reaction mechanism given by Sun et al. for the first time shed light on the collective mechanism [176]. More insightful work is required to look into the reaction behavior at the cathode–electrolyte interface.

Although the aforementioned strategies have proved useful for obtaining a stable cathode–electrolyte interface of LSBs, a versatile method with both economic feasibility and environmental friendliness is still a long haul. The novel vision of the cathode–electrolyte interface still needs further exploration. The good news is that the fundamental issues at the interface have drawn more and more attention. It is believed that, with the ongoing development and strides of electrochemistry and material science, the cathode–electrolyte interface issues will be eventually optimized and the stable operation of LSBs can be prolonged and tap their full potential for wide commercialization.
